# NIR-II Photoresponsive Magnetoliposomes for Remote-Controlled Release and Magnetic Resonance Imaging

**DOI:** 10.1021/acsabm.5c00233

**Published:** 2025-05-22

**Authors:** Laura Fernández-Méndez, Yilian Fernández-Afonso, Pablo Martínez-Vicente, Ainhize Urkola-Arsuaga, Claudia Miranda-Pérez de Alejo, Irati L. de la Pisa, Sandra Plaza-García, Jesús Ruíz-Cabello, Pedro Ramos-Cabrer, Lucía Gutiérrez, Susana Carregal-Romero

**Affiliations:** † Center for Cooperative Research in Biomaterials (CIC biomaGUNE), 540869 Basque Research and Technology Alliance (BRTA), Donostia 20014, Spain; ‡ Euskal HerrikoUnibertsitatea (UPV/EHU), Donostia 20018, Spain; § Instituto de Ciencia de Materiales de Madrid (ICMM/CSIC), Madrid 28049, Spain; ∥ Instituto de Nanociencia y Materiales de Aragón (INMA), CSIC-Universidad de Zaragoza, Zaragoza 50018, Spain; ⊥ Departamento de Bioquímica y Biología Molecular y Celular, Universidad de Zaragoza, Huesca 22002, Spain; # Ikerbasque, Basque Foundation for Science Ikerbasque, Bilbao 48013, Spain; ∇ Centro de investigación en red de enfermedades respiratorias (CIBERES), Instituto de Salud Carlos III, Madrid 28029, Spain; ○ Departamento de Química en Ciencias Farmacéuticas, Universidad Complutense de Madrid, Madrid 28040, Spain; ◆ Centro de Investigación Biomédica en Red de Bioingeniería, Biomateriales y Nanomedicina (CIBER-BBN), Zaragoza 50019, Spain

**Keywords:** magnetoliposome, thermoresponsive, photoresponsive, magnetic resonance imaging, remote controlled release

## Abstract

Magnetic nanoparticles, especially iron oxide nanoparticles, have become versatile and widely used tools in nanomedicine due to their unique magnetic properties, biocompatibility, and tunable functionality. Liposomes have further enhanced the potential of iron oxide nanoparticles by serving as effective nanocarriers with advantages such as drug coencapsulation and enhanced molecular imaging properties. In this study, we present magnetoliposomes composed of ultrasmall free-floating iron oxide nanoparticles inside liposomes (LP-IONPs) and thermoresponsive phospholipids, which were designed as dual *T*_2_-*T*_1_ magnetic resonance imaging (MRI) contrast agents for image-guided liposome degradation and infrared light-responsive nanocarriers in the second biological window for remote-controlled drug delivery. We demonstrated a dynamic shift from *T*_2_ to *T*_1_ MRI contrast during intracellular degradation of LP-IONPs, along with successful light-activated drug release in cancer cells. Biodistribution studies using MRI and histological analysis confirmed their potential for *in vivo* applications. These results highlight the potential of LP-IONPs as image-guided and remote-controlled drug delivery systems.

## Graphical abstract

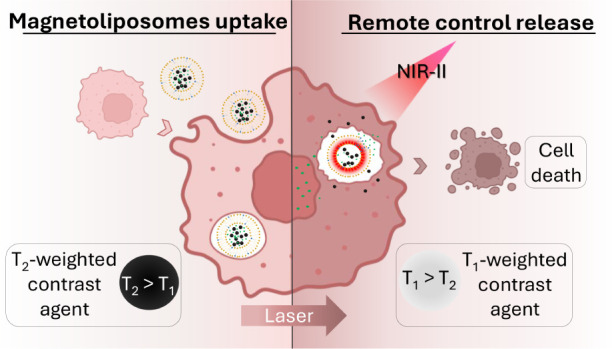

## 1 Introduction

Magnetic nanoparticles (MNPs), especially those based on metals such as iron and its oxides, have become pivotal in nanomedicine.^1,2^ They are extensively used in a multitude of applications, including hyperthermia, nanocatalysis, drug delivery, diagnostics, and theranostics.^3–5^ Advances in nanotechnology have enabled the precise synthesis and functionalization of MNPs, ensuring biosafety and expanding their applications to include clinical use, for example, as magnetic resonance imaging (MRI) contrast agents.^6^

The biosafety profile, magnetic properties, MRI contrast, and specific absorption rate (SAR) of iron oxide nanoparticles (IONPs) can be finely tailored by controlling their composition, morphology, and surface coating.^7–9^ Additionally, the entrapment or nanoconfinement of IONPs in other functional materials, such as poly­(l-lactic-*co*-glycolic acid) (PLGA), micelles, or liposomes, has expanded their functional properties for advanced biomedical applications. These include MRI-guided drug delivery,^10^ transplantation of neural stem cells,^11^ immune stimulation in dendritic cells,^12^ synergistic cancer therapies,^13^ or remotely controlled drug delivery via alternating magnetic fields (AMF).^14^

Liposomes (LPs) stand out as IONP nanocarriers due to their numerous functional advantages, such as straightforward drug coencapsulation, sustained drug delivery, biocompatibility, and biodegradability.^15^ From a structural perspective, they are also advantageous for the entrapment of IONPs in various positions within the LP structure, including the outer shell, the bilayer lipid membrane, or the hydrophilic inner cavity.^14^ The specific location, morphology, and concentration of IONPs in the LPs have been demonstrated to exert a profound influence on the integrity of the LP membrane, the efficiency of drug delivery, the enhancement of MRI contrast, and the heating efficiency under AMFs. However, little is known about the photothermal behavior and drug release ability of IONP-based magnetoliposomes. Recently, IONPs have been applied to near-infrared (NIR) light-triggered hyperthermia, generating heat in their environment that can be used either to stimulate cell death or to achieve remote drug delivery.^16,17^ This rivals the gold-standard application of plasmonic nanoparticles in this field,^18^ so it is worthwhile to analyze the advantages and disadvantages of using IONPs to generate photoresponsive liposomes with inherent MRI imaging properties.

In this study, we developed magnetoliposomes (LP-IONPs) composed of IONPs and thermoresponsive phospholipids, designed as dual *T*_2_-*T*_1_ responsive MRI contrast agents for image-guided liposome degradation and near-infrared (NIR) light-responsive nanocarriers for remotely controlled drug delivery. We demonstrated that the intracellular uptake of LP-IONPs led to a temporal shift from *T*_2_ to *T*_1_ MRI contrast, likely due to liposome degradation, and that remote drug delivery was successfully achieved in cancer cells upon NIR irradiation. Finally, we investigated the biodistribution of LP-IONPs *in vivo* by using MRI and histological analyses.

These magnetoliposomes hold potential for future applications in image-guided drug delivery and remote-controlled release, particularly in settings that combine MRI and NIR light irradiation. Future research should aim to optimize their photothermal efficiency and target tissue accumulation while investigating their interactions with biological systems to expand their range of applications.

## 2 Experimental Section

### 2.1 Chemicals

1–2-Dipalmitoyl-*sn*-glycero-3-phosphocholine (DPPC, #63–89–8), 1-stearoyl-2-hydroxy-*sn*-glycero-3-phosphocholine (Lyso-PC, #9420-57-6), and 1,2-distearoyl-*sn*-glycero-3-phosphoethanolamine-*N*-[methoxy­(polyethylene glycol)-2000] (DSPE-PEG, #880120P) were purchased from Avanti Polar Lipids. 3,3′-Dioctadecyloxacarbocyanine perchlorate (DiOC_18_, #D275) was obtained from Molecular Probes. Iron­(III) chloride hexahydrate (FeCl_3_ 6H_2_O, #236489), hydrazine monohydrate (#207942), doxorubicin (#110603), methanol (#322415), (dimethylthiazol-2-yl)-2–5-diphenyl tetrazolium bromide (MTT, #M2128), Triton X-100 (#T8787), and agarose (#A9539) were acquired from Sigma-Aldrich. PD-10 columns (no. 17085101) were purchased from GE Healthcare. Dimethyl sulfoxide (DMSO) was obtained from Applichem. Polycarbonate membrane filters (110603) were obtained from Whatman PLC. Finally, rhodamine phalloidin (#15119325), ActinGreen 488 (#14879680), calcein AM (#65–0853–78), 4′,6-diamidino-2-phenylindole (DAPI, #10184322), and DMEM FluoroBrite (#15291866) were purchased from Thermo Fisher Scientific.

### 2.2 Synthesis of LP-IONP

Iron oxide nanoparticles (IONPs) were synthesized using the microwave-assisted method developed by Pellico et al.^9^ Magnetoliposomes were synthesized using the thin-film hydration method as described elsewhere.^19,20^ A lipid mixture consisting of 1,2-dipalmitoyl-*sn*-glycero-3-phosphocholine (DPPC) with a molar fraction (*x*) of 0.9, and 1-stearoyl-2-hydroxy-*sn*-glycero-3-phosphocholine (Lyso-PC) with a molar fraction (*x*) of 0.1, was used. Lipids were dissolved in a chloroform/methanol mixture (6:1). The organic solvent was evaporated using a rotary evaporator under high vacuum at 30 °C, resulting in the formation of a thin lipid film, which was further dried under a nitrogen flow for 1 h. The dried lipid film (15 μmol) was rehydrated with 3 mL of IONP at a concentration of 1 mg of Fe/mL, followed by 10 freeze–thaw cycles. The resulting liposome suspension was then extruded at 45 °C using polycarbonate membrane filters with decreasing pore sizes: 400 nm (×2), 200 nm (×4), and 100 nm (×8). After extrusion, the liposomes were purified using a Vivaspin filter with a molecular weight cutoff of 300 kDa, followed by centrifugation at 14,000 rpm for 2 h. The supernatant was carefully removed, and the resulting pellet was resuspended in HPLC-grade water and stored at 4 °C for further characterization. For drug loading, doxorubicin was incorporated into the liposomes during the thin-film formation step. Specifically, 12 mg/mL of hydrophobic doxorubicin in methanol was added to the lipid mixture prior to solvent evaporation, as has been previously reported.^21^

### 2.3 LP-IONP Characterization

The total lipid concentrations of the various liposomal formulations used in this study were quantified using the method of Rouser et al.^22^ Transmission electron microscopy (TEM) was employed to image IONPs and determine their core diameter using a JEOL JEM 2100F microscope operating at 120 kV. Cryo-TEM images of LP-IONPs were also obtained with the same microscope, utilizing a GATAN Model 626 cryotransfer sample holder. The hydrodynamic diameter and electrophoretic mobility of the samples were measured using a Zetasizer Nano ZS instrument (Malvern, Worcestershire, UK). For size determination, IONPs were diluted to a concentration of 50 μg/mL, while liposome samples were diluted to 0.65 μmol/mL phospholipids in HPLC-grade water. ζ-Potential measurements were conducted at the same sample concentrations used for size analysis. Iron content (Fe) was quantified by using inductively coupled plasma mass spectrometry (ICP-MS) with a Thermo Fisher iCap-Q instrument after digestion with nitric acid.

### 2.4 Magnetic Relaxivity Characterization

The spin–lattice (*T*_1_) and spin–spin (*T*_2_) relaxation times were measured for various concentrations of IONPs and LP-IONPs by using a Bruker Minispec MQ60 contrast agent analyzer at 1.5 T and room temperature (RT). For *T*_1_ measurements, a 12-point fitting approach was applied, with repetition times ranging exponentially from 1 ms to 5 × *T*_1_ ms. Additionally, *T*_1_ and *T*_2_ measurements were conducted using a 7 T horizontal bore Bruker Biospec USR 70/30 MRI system (Bruker Biospin GmbH, Ettlingen, Germany). Relaxivity values were calculated by fitting 1/*T*_1_ or 1/*T*_2_ (s^–1^)) against the iron (Fe) concentration in the samples (mM) using Origin 9.8 software.

### 2.5 Light Absorbance Measurements

The absorbance of LP-IONPs and IONPs was characterized using a UV–vis-NIR spectrophotometer (Jasco V670). A 100 μL suspension was placed in a quartz cuvette with a 3 mm optical path length. Spectra were recorded over a wavelength range of 800–1200 nm. The sample concentration was 0.6 mg of Fe/mL.

### 2.6 Magnetic Measurements

Sample preparation for magnetic measurements was performed by placing a volume (150 μL) of the particle’s suspension ([Fe] = 1.3 or 0.6 mg/mL for IONP and LP-IONP) into a piece of cotton wool and allowing it to dry at room temperature. The dried wool was then placed inside a gelatin capsule for magnetic characterization. Additionally, a very diluted sample of the IONPs was dispersed in agar, solidifying the material while being placed in an ultrasonic bath to allow for a homogeneous dispersion of the IONPs. This diluted sample was also freeze-dried and used for magnetic characterization by placing the lyophilized sample in a gelatin capsule. Magnetic measurements were performed in a Quantum Design (USA) MPMS-XL SQUID magnetometer. Field-dependent magnetization was recorded at 300 K in the field ranges between −2000 and 2000 kA/m. AC magnetic susceptibility measurements were performed with a field amplitude of 326 A/m and a frequency of 11 Hz in the temperature range from 2 to 40 K.

### 2.7 Photothermal Conversion

The photothermal conversion was studied by measuring the temperature variation of the solution under exposure to NIR light. The nanoparticle suspension (*V* = 0.5 μL at 0.6 mg Fe/mL) was placed into a quartz cuvette (2 mm optical path) with magnetic stirring. The cuvette containing the sample was irradiated for 5 min using a laser (Laser Quantum, mpc6000/Ventus 1064) of λ = 1064 nm and a power of 1 W, while recording the suspension temperature with a thermocouple (T-type) coupled to a Datalogger USB (TC Direct). The beam spot size was 2.2 mm (beam diameter), resulting in a very small, irradiated area (0.04 cm^2^)) and an irradiated sample volume of 8 μL. The power density (power per unit area) was 26 W/cm^2^. The high power density was necessary for particle characterization to achieve sufficient heating for detection, given the configuration of our system. Within the cuvette, a very small spot size is irradiated, so only a few particles are exposed to the laser. However, a magnetic stirrer continuously moves the colloidal suspension, ensuring that different particles are irradiated over time.

### 2.8 Thermal and Photothermal Release of DOXO

To evaluate the photothermally triggered release of doxorubicin (DOXO) from the LP-IONP_DOXO_, aliquots of nanoparticles were dispensed into Eppendorf tubes containing cell culture medium at a final concentration of 0.46 mg of Fe per mL of cell medium. First, samples were incubated for 10 min at three distinct temperatures (30 °C, 37 °C, and 45 °C) and centrifuged for 10 min at 2000 rpm. Subsequently, the supernatant was collected, and the fluorescence of DOXO was measured. The release of DOXO from LP-IONP_DOXO_ was also evaluated following NIR-II light irradiation (1064 nm). For this, LP-IONP_DOXO_ was placed in a 96-well plate and continuously irradiated with 1 W power for 10 min. After irradiation, samples were centrifuged (2000 rpm, 10 min), and the supernatant fluorescence was measured. In both cases, the supernatant’s fluorescence was measured using a Biotek Synergy H1 microplate reader with an excitation wavelength of 480 nm and an emission wavelength of 590 nm.^23^

### 2.9 Viability and Cell Uptake Assays

First, we conducted an analysis to evaluate the effect of LP-IONP on cellular mitochondrial activity. This was achieved by performing a 3-(4,5-dimethylthiazol-2-yl)-2,5-diphenyl tetrazolium bromide (MTT) assay in two different cell cultures: HepG2 hepatic cells and MDA-MB-231 breast epithelial cells. Finally, the absorbance of the samples was measured at 550 nm by using a microplate reader (Biotek Synergy H1 microplate reader). The data obtained from the assay were used to plot cell viability graphs, which were generated using GraphPad Prism 9. To allow the visualization of LP-IONP inside cells, DiOC_18_ was added to the lipid mixture before forming the lipid film. Two cell lines, HepG2 hepatic cells and MDA-MB-231 breast epithelial cells, were seeded onto 24-well plates at a density of 2.5 × 10^4^ cells per well and cultured for 24 h. Next, cells were incubated with the different nanoparticles at a concentration of 20 μM for 4, 24, and 48 h at 37 °C and 5% CO_2_. Following incubation, the cells were washed with PBS and fixed with 4% paraformaldehyde (PFA). Cell membranes were stained with Rhodamine phalloidin (1:50 ActinRed, Thermo Fisher Scientific, #15119325) for 45 min. After another PBS wash, the cell nuclei were stained with DAPI at a concentration (1:1000, Thermo Fisher Scientific, #10184322) for 10 min. Finally, cells were washed with PBS and immersed in a mounting medium (DACO, Agelent, #S302380–2), and imaged using a Cell Axio Observer Microscope. The uptake of LP-IONP_DOXO_ was assessed by using DOXO fluorescence as an indicator. The experimental protocol followed was identical to that of LP-IONP, with the addition of an additional time point at 6 h. The colocalization of DOXO with the DAPI staining of the cell nucleus was quantified using the Pearson correlation coefficient. Imaging was conducted using a Cell Axio Observer Microscope and analyzed through ImageJ software, employing the JACoP plugin for its assessment.

### 2.10 Intracellular TEM Evaluation

Cells were washed with 0.1 M PBS and then incubated for 10 min at 37 °C with 3% glutaraldehyde in 0.1 M phosphate buffer (PB) at a ratio of 4:1 of Na_2_HPO_4_ to NaH_2_PO_4_. The glutaraldehyde was removed, and cells were then treated with 3% glutaraldehyde in 0.1 M PB for 1 h at room temperature. Finally, the glutaraldehyde was removed, and the cells were washed 10 times with 0.1 M PB. Cell images were acquired with a high-angle annular dark field detector (STEM-HAADF) using a Tecnai F30 (FEI) microscope with an accelerating voltage of 300 keV. EDX (Energy-dispersive X-ray spectroscopy) analysis was used to evaluate the presence of iron through its characteristic X-ray energies (Kα 6.398 keV and Lα 0.705 keV).

### 2.11 Intracellular MRI Measurements

1.5 million HepG2 cells were seeded overnight in a T-25 flask. Following that, the cells were treated with different nanoparticles, IONP and LP-IONP. Both nanoparticles were used at a concentration of 9 μg Fe/mL. The treatments were carried out for four different time points: 6 h, 24 h, and 3 days. The incubation was conducted at 37 °C, 5% CO_2_, and 95% air. After the respective incubation times, the cells were washed with PBS, treated with trypsin to detach them from the flask surface, and washed. Subsequently, the cell pellet resuspended in 250 μL of PBS was mixed and homogenized with 250 μL of a 1% agarose solution. *T*_1_ and *T*_2_ values were performed on a 7 T Bruker Biospec 70/30 USR MRI system (Bruker Biospin GmbH, Ettlingen, Germany), interfaced to an AVANCE III console. Images were acquired using a 4 cm inner diameter volumetric coil for radio frequency transmission and reception (RX/TX) from Bruker. The ParaVision 7.0 software is also from Bruker.

### 2.12 Photothermal Response of LP-IONP and LP-IONP_DOXO_ *In Vitro*

Magnetoliposomes were incubated with MDA-MB-231 cells for 5 h at a final concentration of 100 μM of phospholipids and 0.8 μmol of Fe/mL. MDA-MB-231 cells were stained with calcein AM (2 μM, 30 min) to allow the visualization of living cells over time. After staining, the cells were washed twice with PBS and replaced with DMEM FluoroBrite (cell media without phenol red and 10% FBS). Subsequently, the cells were irradiated with NIR-II light at a power of 1 W for 10 min). Following irradiation, the cells were placed on the cell observer microscope overnight at 5% O_2_ and 37 °C for the continuous monitoring of cell viability over time.

### 2.13 *In Vivo* Biodistribution Study

All animal studies were performed at CIC biomaGUNE. Animals were maintained and handled in accordance with the Guidelines for the Accommodation and Care of Animals (European Convention for the Protection of Vertebrate Animals Used for Experimental and Other Scientific Purposes). All animal procedures were performed under the European Union Animal Directive (2010/63/EU). Experimental procedures were approved by the local Ethical Committee of CIC biomaGUNE and by the local authorities (Diputación Foral de Guipúzcoa, project number PRO-AE-SS-091). For the biodistribution study (*n* = 4), the dose used was 1.5 mg/kg of Fe. The corresponding experimental protocol can be found in the Supporting Information.

## 3 Results and Discussion

### 3.1 Synthesis and Characterization of LP-IONP

Thermoresponsive magnetoliposomes (LP-IONP) were synthesized using a thin-film hydration method, followed by freeze/thaw cycles and extrusion (Figure 1A).^20^ Citrate-coated iron oxide nanoparticles (IONPs) with a mean hydrodynamic diameter (*d*_h_) of 9.4 ± 1.2 nm and a zeta potential of −19 ± 1 mV (Figure 1B,C) were initially prepared according to the microwave-assisted synthesis protocol developed by Pellico et al., ensuring positive MRI contrast.^9^

**Figure 1:**
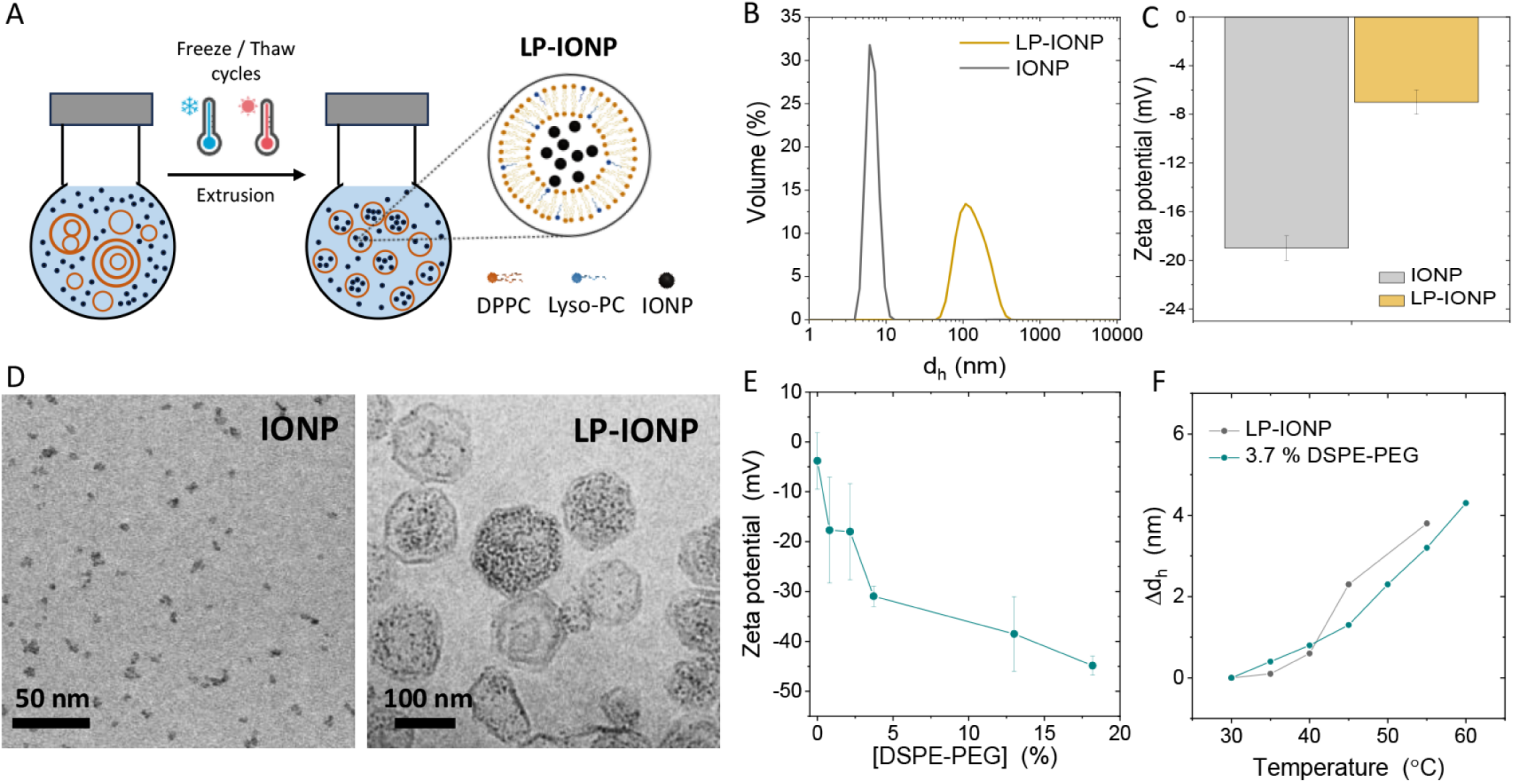
(A) Schematic illustration of the synthesis of LP-IONP. (B) Hydrodynamic diameter (*d*_h_) measurements by DLS, (C) zeta potential of IONP (gray) and LP-IONP (orange). (D) TEM images of IONP. (F) CryoTEM images of LP-IONP. (E) Zeta potential of PEGylated LP-IONP modified with different percentages of DSPE-PEG. (F) Increase in *d*_h_ of the thermoresponsive liposomes with increasing temperature.

To synthesize magnetoliposomes, IONPs (1 mL, 1 mg of Fe/mL) were used to hydrate a phospholipid thin film comprising 5 μmol of 1,2-dipalmitoyl-*sn*-glycero-3-phosphocholine (DPPC) and 1-stearoyl-2-hydroxy-*sn*-glycero-3-phosphocholine (Lyso-PC) at a molar ratio of 0.9:0.1. The hydrated vesicles underwent repeated freeze/thaw cycles to physically disrupt the phospholipid bilayers, effectively breaking apart multilamellar vesicles and creating more unilamellar liposomes with larger internal volumes to entrap IONPs.^24^ Extrusion and purification by filtration were then employed to homogenize the LP size and remove the free IONPs.

The final LP-IONPs had an iron molar ratio of 1.7 ± 0.1 [Fe]/ [phospholipid], a mean *d*_h_ of 154.4 ± 5.3 nm, and a zeta potential of −7 ± 1 mV (Figure 1B,C), similar to empty DPPC:Lyso-PC liposomes (LPs), which had a mean *d*_h_ of 136.3 ± 2.8 nm and a zeta potential of −6 ± 2 mV (Figure S1A). Transmission electron cryomicroscopy (CryoTEM) (Figure 1D) confirmed that the IONPs were predominantly entrapped within the inner cavities of the liposomes.

Furthermore, when LP-IONPs were disrupted with 2% of the nonionic surfactant Triton X-100,^25^ we observed that the entrapment of IONPs in the interior of the LPs resulted in negligible IONP agglomeration. After their release with Triton, the IONPs remained well-dispersed, as shown in the cryoTEM images of Figure S1B. These images of LP-IONPs before and after LP disruption, along with the corresponding size measurements, confirmed that LP-IONPs had a diameter of *d*_TEM_ = 126 ± 36 nm before disruption, while the released IONPs measured *d*_TEM_ = 4 ± 1 nm after disruption, confirming their free-floating status within the LP. The colloidal stability of LP-IONPs was evaluated in water and in media containing serum over a 5-day incubation period. As shown in Figure S1C, the addition of 0.1% fetal bovine serum (FBS) had no effect on the *d*_h_. However, at 1% FBS, an increase in *d*_h_ was observed at short incubation times, likely due to the protein corona formation. While polydispersity also increased under these conditions, no statistically significant differences in *d*_h_ were detected between LP-IONPs in water and those in serum-containing solutions after 5 days of incubation.

Since many liposomes designed for intravenous administration include pegylated phospholipids in their composition due to their antifouling effect and the increase of colloidal stability *in vivo*,^26^ we studied whether the lipid composition of the LP-IONPs could be modified to incorporate 1,2-distearoyl-*sn*-glycero-3-phosphoethanolamine-*N*-[amino­(polyethylene glycol)-2000] (DSPE-PEG) or other functional phospholipids. Incorporating DSPE-PEG during the thin-film formation step resulted in negligible IONP loading (Figure S1C), likely due to repulsive or steric hindrance interactions due to the presence of this 2000 Da PEG in the formulation.

To address this, we used a simple postmodification protocol in which LP-IONPs were incubated with diluted DSPE-PEG in a warmed aqueous solution under magnetic stirring.^27^ High-performance liquid chromatography–mass spectrometry (HPLC-MS) confirmed the successful incorporation of DSPE-PEG with the addition of increasing volumes of DSPE-PEG, achieving up to 18% molar ratio. This modification induced a progressive decrease in the zeta potential, reaching −45 ± 2 mV (Figure 1E). Notably, the LP-IONPs retained their thermoresponsive swelling behavior after PEGylation (Figure 1F) with no loss of IONPs or colloidal stability observed during this surface modification process. This approach can serve as a strategy for incorporating functional ligands into thermosensitive magnetoliposomes, thereby expanding their potential applications.

### 3.2 Characterization of LP-IONP as Switchable MRI Contrast Agents

To evaluate the potential of LP-IONPs as MRI contrast agents, we measured their longitudinal (*T*_1_) and transverse (*T*_2_) relaxation times at increasing iron concentrations using both a minispec (1.5 T) and a 7 T MRI scanner (Figure 2). For comparison, we also assessed these relaxation times for IONPs prior to liposomal encapsulation to investigate how encapsulation influenced their performance as *T*_1_ and *T*_2_ contrast agents. As shown in Figure 2A,B, the encapsulation of IONPs within liposomes significantly enhanced their T_2_-shortening effects. Typically, lower *r*_2_/*r*_1_ values (1 ≤ *r*_2_/*r*_1_ ≤ 3) are associated with *T*_1_ contrast agents, while higher values (*r*_2_/*r*_1_ > 10) are characteristic of *T*_2_ contrast agents. Our results at 1.5 T confirmed that encapsulating IONPs, which behave as *T*_1_ contrast agents (Figure 2B, *r*_1_= 7.0 ± 0.1 mM^–1^ s^–1^, *r*_2_= 17.2 ± 0.3, and *r*_2_/*r*_1_= 2.4), led to the generation of highly efficient *T*_2_ contrast agents in the form of LP-IONPs (Figure 2A, *r*_1_= 14.9 ± 0.4 mM^–1^ s^–1^, *r*_2_= 232 ± 13 mM^–1^ s^–1^, and *r*_2_/*r*_1_= 15.6 at 1.5 T). This enhancement can be partially attributed to the increased concentration of IONPs within the interior of the liposomes, as previously observed in liposomes with higher encapsulated IONP concentrations.^28^ Additionally, the interaction, whether constructive or destructive, between the magnetic fields induced by individual IONPs with *T*_1_ contrast could create a more complex magnetic environment, accelerating the relaxation process of nearby water protons.^29^ These results are consistent with the relaxation time shortening of *T*_1_ and *T*_2_ obtained at 7 T in an MRI scanner for both IONPs and LP-IONPs (Figure 2B).

**Figure 2:**
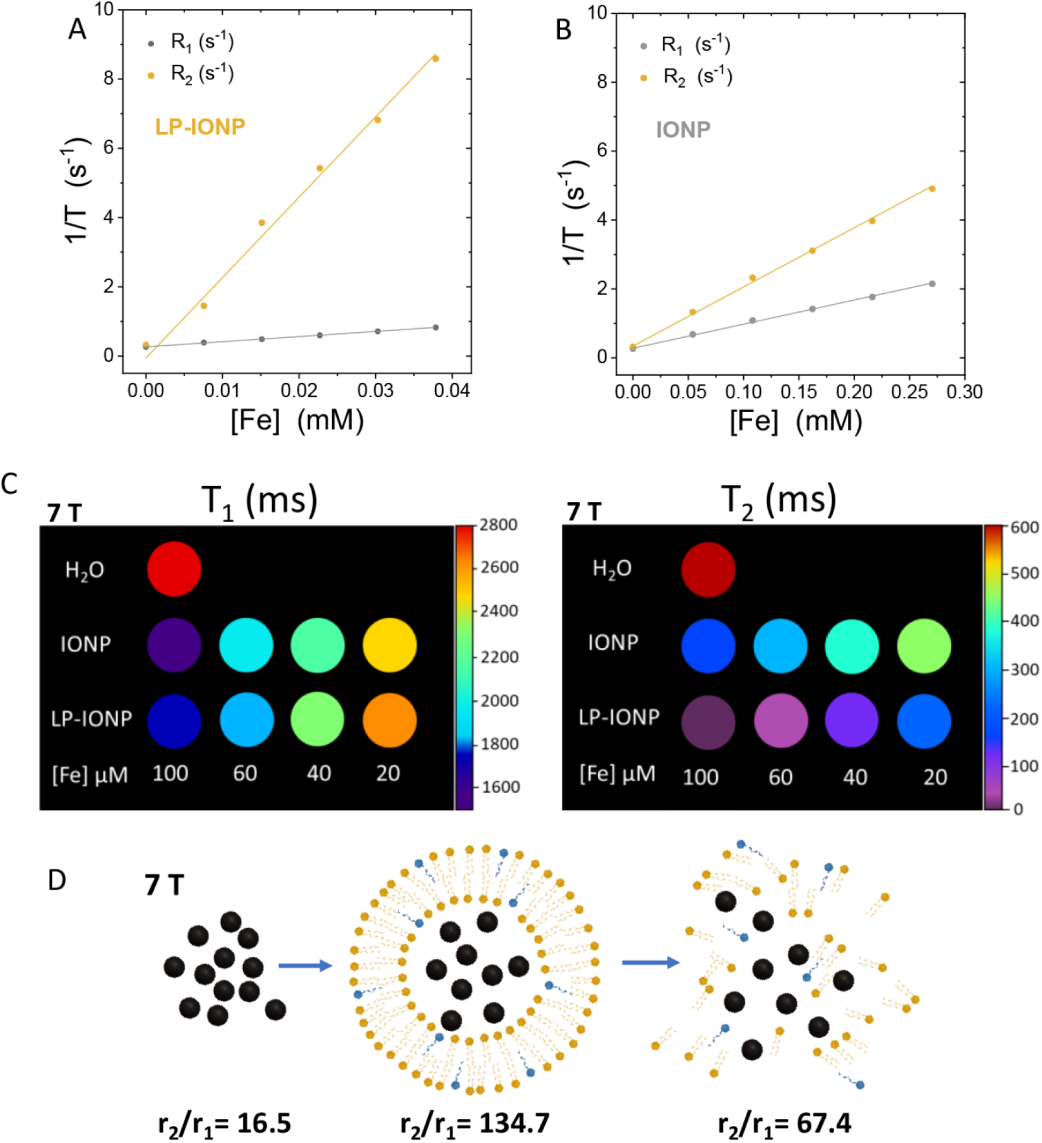
*T*_1_ and *T*_2_ relaxation rates of LP-IONP (A) and IONP (B). (C) 7 T MRI contrast of LP-IONP compared with IONP at different iron concentrations. (D) Schematic illustration of the LP-IONP disruption with Triton X-100 and the corresponding *r*_2_/*r*_1_ values obtained with a 7T MRI scanner.

Disruption of LP-IONPs with Triton X-100, as shown in Figures 2D and S1B, induced the release of IONPs, shifting the MRI contrast back toward *T*_1_ contrast agent behavior (*r*_1_= 9.2 ± 0.1 mM^–1^ s^–1^, *r*_2_= 26.6 ± 2.9 mM^–1^ s^–1^, and *r*_2_/*r*_1_= 2.7 at 1.5 T). This is consistent with Triton X-100’s ability to partially release liposomal cargo, which explains the partial recovery of the relaxometric properties of free IONPs.^30^ However, a minor degree of IONP clustering cannot be ruled out, despite no such aggregation being observed in the cryo-TEM images.

These findings are particularly significant, as hybrid nanomaterials based on liposomes that can switch between *T*_1_ and *T*_2_ contrasts are scarce. Previous studies have explored the coencapsulation of gadolinium-based *T*_1_ contrast agents and IONPs within liposomes as a strategy to achieve this type of MRI contrast modulation.^31^ In our study, we demonstrate that for LP-IONPs, it is sufficient to encapsulate IONPs smaller than 5 nm with T_1_ contrast, provided they exhibit adequate colloidal stability to prevent agglomeration during the encapsulation process. Notably, previous research involving the encapsulation of 16 nm IONPs within liposomes to enhance MRI contrast showed that while shifts in *r*_2_/*r*_1_ values could be achieved with high amounts of encapsulated IONPs, the resulting magnetoliposomes consistently retained the T_2_ contrast character upon liposome disruption.^28^

### 3.3 Magnetic Properties and Interparticle Interactions in LP-IONPs

To better understand the effect of encapsulating iron oxide nanoparticles (IONPs) within our thermoresponsive liposomal formulations (LP-IONPs), we investigated their magnetic properties. Both IONP and LP-IONP exhibited superparamagnetic behavior at room temperature, as evidenced by their negligible remanence and coercivity (Figure 3A). The saturation magnetization value (Ms) was 57 Am^2^/kg of Fe_3_O_4_ for LP-IONP and 42 Am^2^/kg of Fe_3_O_4_ for IONP (Figure 3A inset). This value for IONP, while lower than that of bulk magnetite, aligns with the expectations for magnetite nanoparticles of this size.^32^ The increase in magnetic susceptibility observed in the LP-IONP sample (slope of d*M*/d*H* at low magnetic fields) suggests more cooperative behavior due to the increase in interactions between the nanoparticles within the liposomes.

**Figure 3:**
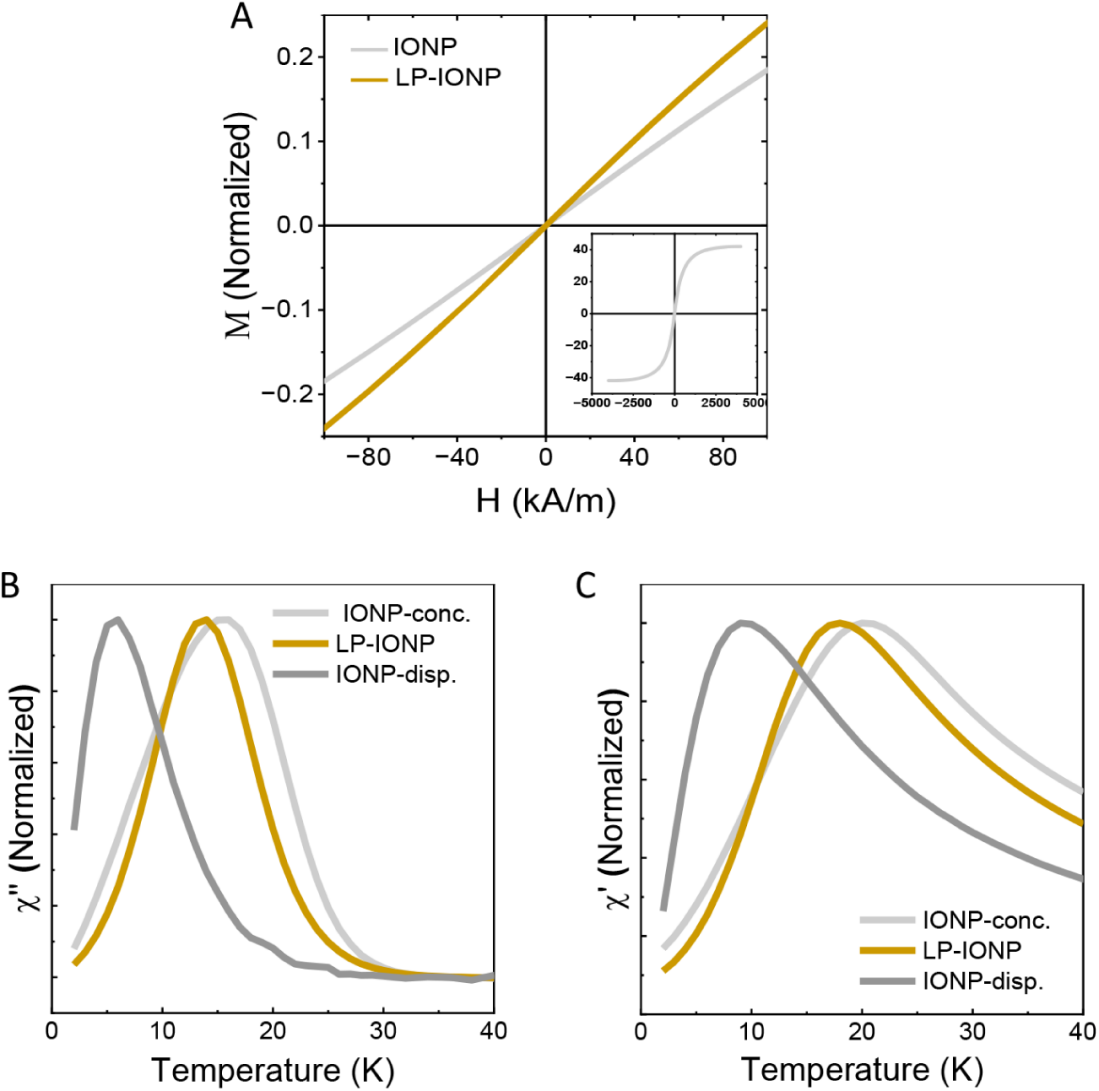
Magnetic measurements. (A) Field-dependent magnetization at room temperature of the IONP and LP-IONP samples. (B, C) Temperature dependence of the two components of the AC magnetic susceptibility (out-of-phase (χ″) and in-phase (χ′)) scaled to their maxima of the IONP concentrated in cotton (conc.) or dispersed in agar (disp.) and the LP-IONP samples.

To further evaluate the aggregation of the particles within the liposomes, we studied the interparticle dipolar interactions by analyzing the temperature dependence of the AC magnetic susceptibility (Figure 3B,C). We prepared two IONP systems with distinct levels of dipolar interactions: one by drying and concentrating the IONP suspension on cotton, inducing strong particle interactions, and the other by dispersing the IONPs in agar, minimizing interparticle interactions. A marked difference emerged between these two systems; the temperature of the out-of-phase susceptibility maximum shifted significantly (Figure 3C) from 6 K for the diluted system to approximately 16 K for the aggregated sample. This shift, associated with increasing dipolar interactions, is a well-documented phenomenon in magnetic nanoparticles.^33^

For LP-IONPs, the out-of-phase susceptibility maximum was observed at 14 K (Figure 3C), indicating a stronger degree of dipolar interactions compared to that of the agar-dispersed IONPs. This behavior is consistent with the confinement and high loading of IONPs within the liposomes, as corroborated by the cryo-TEM images (Figure 1D).

### 3.4 Thermal and Photothermally Induced Drug Delivery

The phospholipid composition of LP-IONP (DPPC:Lyso-PC) was selected because it is similar to that of ThermoDox, a thermosensitive liposome-based formulation of the anticancer drug doxorubicin (DOXO). ThermoDox enables the localized release of high concentrations of DOXO in response to increased temperature (39–42 °C) and has progressed to Phase III clinical trials in recent years.^34^ These trials combined ThermoDox with radio frequency ablation for liver cancer treatment, aiming to achieve controlled DOXO release and an enhanced antitumoral effect. However, the results showed no significant advantage over free DOXO, leading to the discontinuation of the trials.

Despite these outcomes, alternative physical stimuli such as alternating magnetic fields or light could expand the potential applications of similar thermosensitive liposomes, even under mild hyperthermia conditions.^35^ In our study, we incorporated ultrasmall IONPs into these thermoresponsive liposomes, creating LP-IONP, and demonstrated their switchable MRI contrast capability. We then investigated whether the presence of IONPs affected the thermoresponsive properties and DOXO release of the liposomes and if NIR light can trigger the release of DOXO (Figure 4A).

**Figure 4:**
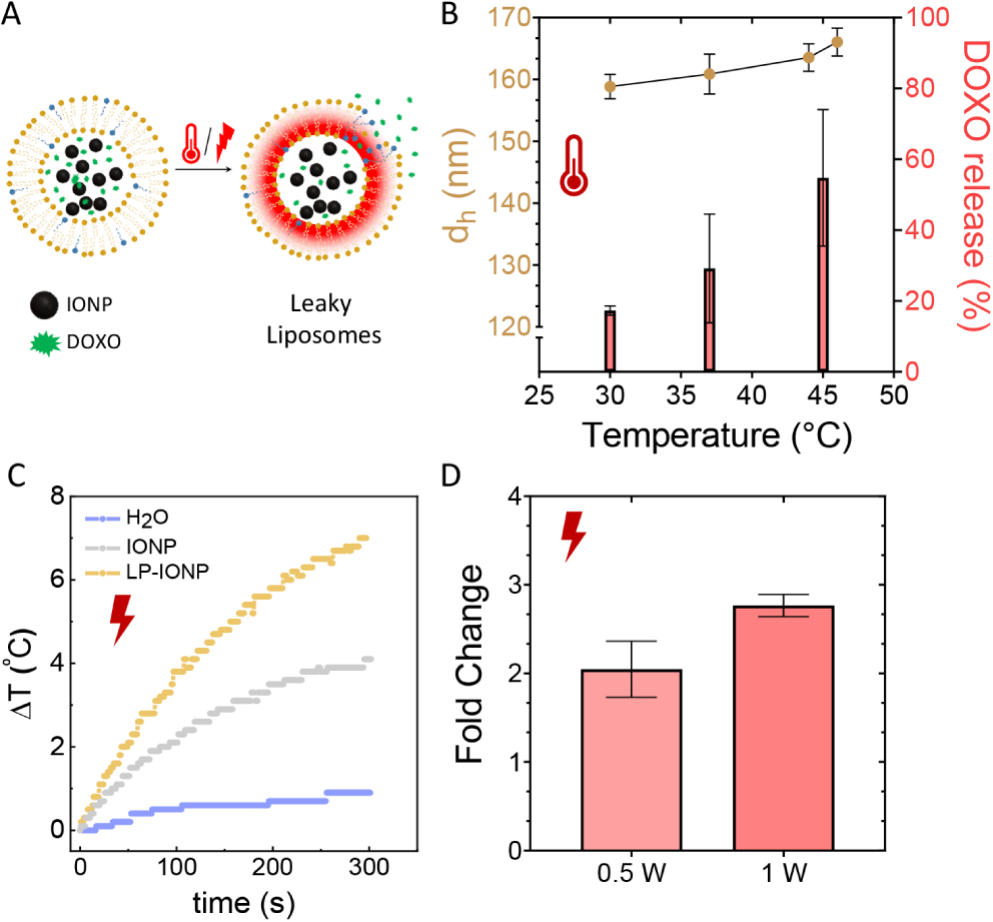
(A) Schematic illustration of DOXO release triggered by pore formation in the lipid bilayer induced by either temperature increase or NIR-II light irradiation. (B) Percentage of DOXO released at different temperatures, along with the corresponding hydrodynamic diameter (*d*_h_) of LP-IONPs. (C) Temperature increases in LP-IONP and free IONP solutions compared to water under NIR-II light exposure. (D) Fold change in DOXO release (35–48%) from LP-IONP_DOXO_ solutions (0.46 mg Fe/mL) in cell media after 10 min of exposure to various laser powers, relative to nonirradiated LP-IONP_DOXO_ incubated at 37 °C for 10 min.

To assess this, we coencapsulated DOXO and IONPs inside the liposomes (LP-IONP_DOXO_) and first measured the DOXO release at different temperatures after 10 min of incubation in cell media, correlating it with changes in hydrodynamic diameter to confirm that heat did not compromise colloidal stability (Figure 4B).

The mechanism of rapid drug release from thermoresponsive liposomes composed of DPPC:Lyso-PC, similar to LP-IONP, at temperatures between 39 and 45 °C was previously described by Needham et al.^36^ They proposed that heating to the lipid transition temperature induces the formation of leaky interfacial regions or pores within the lipid bilayer. Lyso-PC plays a crucial role in stabilizing these pores, thereby facilitating efficient drug release. Notably, this mechanism allows for the rapid release of DOXO within seconds.^37^

A direct comparison between the previously reported DOXO release of DPPC:Lyso-PC liposomes and our LP-IONP showed that approximately 75% of DOXO was released within 10 min at 45 °C in cell media, whereas our LP-IONP_DOXO_ released nearly 60% in cell media and 80% in water (Figure S2). This indicates a slight reduction in DOXO release efficiency in complex biological media. However, the observed DOXO release of LP-IONP can be considered similar to other reported thermoresponsive liposomes designed for drug delivery, despite the presence of the IONP inside the liposome.^38^

Hydrodynamic diameter measurements (Figure 4B) revealed that, alongside the formation of pores facilitating the rapid release of DOXO, the liposomes underwent slight swelling while maintaining their colloidal stability. This effect is not usually taken into account but may indicate an increase in membrane fluidity and, therefore, increased DOXO leakage.

To assess the photothermal properties of LP-IONP_DOXO_, we irradiated solutions containing known concentrations of iron (0.6 mg/mL) in both LP-IONPs and free IONPs, using NIR light within the second biological window (wavelength 1064 nm and 1 W laser power). The resulting temperature increase over time was monitored and is shown in Figure 4C. NIR light within the second biological window (1000–1350 nm) penetrates tissue more deeply (from 1–6 mm to 20 mm approximately) than light in the first biological window (NIR-I, 650–900 nm).^39^ Furthermore, Fe_3_O_4_ NPs are known to exhibit significantly higher optical absorption within this range,^40^ as we also observed for free IONPs, but was not evident for the liposomal formulation due to scattering.

The results demonstrated that LP-IONPs exhibited a superior ability to increase the temperature of the surrounding water compared with free IONPs. Subsequently, we calculated the specific loss power (SLP) and photothermal conversion efficiency (η) for both LP-IONPs and free IONPs using eqs 1 and 2, respectively.

1 $$\eta =\frac{m_{H_{2}O}\mathrm{SLP}}{I\left(1-10^{-A}\right)}$$

2 $$\mathrm{SPL}=\frac{c_{H_{2}O}m_{H_{2}O}}{m_{Fe}}{\frac{dT}{dt}|}_{t\to 0}$$

Where $C_{H_{2}O}$ is the specific heat capacity of water (4.186 J g^–1^ K^–1^), $m_{H_{2}O}$ is the mass of water (g), *m*_Fe_ is the mass of Fe (g) corresponding to NPs in the irradiated volume (considering the spot size and the cuvette length), and (d*T*/d*t*) is the initial slope of the heating curve (first 30 s). *I* (1 W) is the laser power incident in the sample and *A* is the absorbance of the sample at the irradiation wavelength.

The SLP values for LP-IONP and IONP were determined to be 20.9 and 13.9 kW/g, respectively, indicating an enhancement in the heating capacity of the particles upon encapsulation. Using these values, the photothermal efficiency was calculated based on eq 2. Due to the significant differences in absorbance (LP-IONP: 0.35 and IONP: 0.05) (Figure S3) between the two samples, the resulting photothermal efficiency was higher for free IONP (η = 0.49) compared to LP-IONP (η = 0.17). These efficiencies fall within the range of previously reported values in the literature.^41–43^ However, direct comparison with other studies remains challenging due to variations in measurement conditions, experimental setups, and data analysis methodologies.

The results demonstrate that encapsulating IONPs in LP-IONPs significantly enhances the system’s heating capacity, which is a critical factor for DOXO release under NIR-II light irradiation. As shown in Figure 4D, DOXO release from the LP-IONP_DOXO_ sample increased by approximately 2- to 2.7-fold after 10 min of NIR-II irradiation compared to the nonilluminated sample incubated at 37 °C. Furthermore, this light-triggered drug release was strongly dependent on laser power, with higher power levels resulting in greater DOXO release, as illustrated in Figure 4D.

The observed DOXO release aligns with previously reported values for liposomes with diameters between 230 and 250 nm, composed solely of DPPC and DSPE-PEG, loaded with hydrophobic IONPs (30 nm) embedded within the phospholipid bilayer.^44^ These systems, irradiated for 10 min at 885 nm (0.7 W, spot size: 5 × 8 mm^2^), demonstrated a 2-fold increase in DOXO release compared to nonirradiated samples and achieved phase transition temperatures as high as 47 °C. In contrast, our magnetoliposomes, which incorporate Lyso-PC to achieve lower transition temperatures (∼41.3 °C),^45^ offer several distinct advantages: reduced thermal thresholds to avoid excessive heating, lower amounts of IONPs to minimize the risk of iron accumulation in tissues, and functionality as MRI contrast agents with switchable contrast capabilities.^46^

Additionally, our system employs irradiation in the NIR-II window, enabling deeper tissue penetration compared to the NIR-I window, thereby enhancing its potential for clinical applications. Furthermore, because the lipid bilayer in our system was not directly loaded with IONPs, the structural integrity of the bilayer was better maintained, preserving the stiffness and flexibility of the liposomes. These properties are critical, as they influence cell uptake, half-life elimination, tumor accumulation, and tumor penetration of the liposomes, further highlighting the advantages of our LP-IONP.

Our results for laser-induced DOXO release were also comparable to those obtained with warmed solutions of thermosensitive liposomes. For instance, Mills et al. reported a 4-fold increase in DOXO release between 37 °C and mild heating at 40 °C in DOXO-loaded liposomes containing 10% monostearoylphosphatidylcholine (MSPC), DPPC, and DSPE-PEG (4%).^36^ Similarly, the application of alternating magnetic fields (AMF), which generate heat more homogeneously than NIR irradiation,^47^ has been shown to induce comparable DOXO release (approximately 40–50%) at similar iron concentrations (∼0.6 mg Fe/mL).^48,49^ Each thermal trigger presents distinct advantages: laser-based photothermal release enables precise spatial control, tunable energy input, and compatibility with a broader range of materials, making it particularly suitable for localized drug delivery with minimal off-target effects.^50^ Conversely, AMF-induced hyperthermia provides uniform heating throughout the sample volume but offers less spatial resolution and material versatility. Taken together, these comparisons underscore the relevance of NIR-II-mediated release as a promising alternative to conventional hyperthermia, offering spatial precision and energy efficiency.^50^ These similarities in DOXO release underscore the effectiveness of our system for controlled drug delivery. However, the localized heat generated by the encapsulated IONPs and the subsequent heat dissipation within our liposomes require more precise analysis using nanothermometry tools.^51^ Such studies would enable a more direct comparison to other photoresponsive liposome systems.

### 3.5 Intracellular Switch of MRI Contrast and Light-Triggered Remote-Controlled Release

MRI is a noninvasive diagnostic technique that uses strong magnetic fields and radio waves to generate detailed images of the body’s internal structures, without exposing patients to ionizing radiation. By aligning and then disrupting the orientation of hydrogen protons in tissues, MRI captures the emitted signals as the protons realign, allowing for high-resolution imaging, particularly effective for soft tissues such as the brain, spinal cord, and internal organs.^52^ To investigate both the potential intracellular MRI contrast shifts induced by IONP delivery from cellular endolysosomes after LP-IONP uptake and the capability of remotely triggering DOXO release under NIR-II light exposure, we first assessed the cell viability and uptake in two relevant cell lines exposed to LP-IONP: HepG2 liver cells and MDA-MB-231 breast cancer cells (Figure S4). HepG2 liver cells were selected because liposomes of approximately 100–200 nm in size tend to accumulate in the liver compared to other organs after their intravenous administration.^53^ Additionally, MDA-MB-231 breast cancer cells were chosen due to the potential application of these photosensitive liposomes in antitumor therapies.^54^ The results shown in Figure S4A,B indicate that, in both cell lines, liposomes with phospholipid concentrations up to 150 μM did not affect cell viability, which remained between 90% and 100% across all tested concentrations after 24 h of LP-IONP incubation. At extended exposure times (48 h), a decrease in viability below 80% was observed only at LP-IONP concentrations above 100 μM. To further investigate the potential induction of ferroptosis, a distinct form of regulated, iron-dependent cell death characterized by lipid peroxide accumulation and intracellular glutathione depletion, we conducted incubation assays with LP-IONP for 24, 48, and 72 h. Intracellular thiol levels (primarily glutathione) were measured using the ThiolTracker Violet probe, both in HepG2 and MDA-MB-231 cells. As shown in Figure S4C,E, no significant differences in thiol levels were detected across the different exposure times and concentrations, indicating minimal toxicity due to iron accumulation within the experimental time frame. Confocal microscopy-based internalization studies (Figure S5) revealed that liposomes labeled with the green fluorophore DiOC_18_ accumulated within the perinuclear region of the cell and showed partial cytosolic release after 24 h.

Intracellular shifts in MRI contrast that could correlate with drug delivery were examined in HepG2 cells over 3 days, with the main results presented in Figures 5 and S6. Initially, LP-IONP acted as *T*_2_ contrast agents, as their uptake at 6 h resulted in a predominant *T*_2_ response, reflected by an increase in *R*_2_ and only a slight variation in *R*_1_. After 24 h of incubation, fluorescence imaging showed that DiOC_18_ was distributed both in vesicles within the cytosol and more diffusely throughout the whole cytosolic volume (Figure S5). However, the *T*_2_ response (*R*_2_) remained dominant. This suggests that the release dynamics of IONPs differ (hydrophilic and *d_h_* = 9.4 nm) from those of small hydrophobic molecules, indicating that these magnetoliposomes may not be suitable for monitoring the release of such molecules.

**Figure 5:**
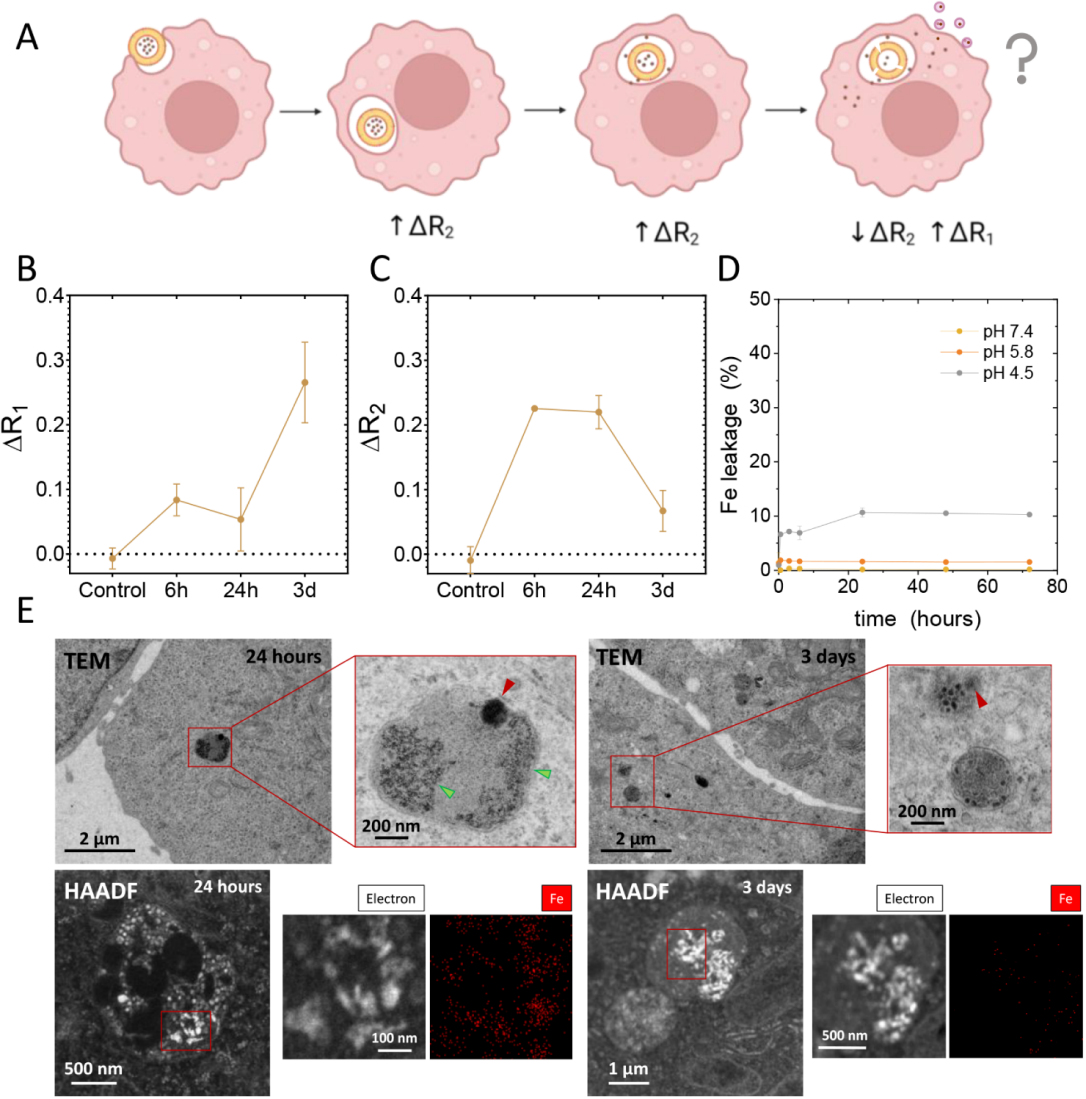
(A) Schematic diagram of the potential intracellular fate of the LP-IONPs. MRI measurements (7 T) showing variations in *R*_1_ (B) and *R*_2_ (C) over time in HepG2 cells incubated with LP-IONPs. (D) Temporal iron leakage from LP-IONP exposed to different pH values over 3 days. (E) Representative TEM and HAADF images of HepG2 cells incubated for 24 h and 3 days with LP-IONP, along with the corresponding EDX image of Fe in selected regions of interest marked with red squares.

By day 3, *R*_1_ increased significantly, indicating the presence of IONPs acting as *T*_1_ contrast agents inside the cells, while the *T*_2_ response (*R*_2_) decreased, pointing to notable changes in IONP concentration and localization within the cells. To elucidate this shift in MRI contrast, we studied the potential dissolution of IONPs under different pH conditions, mimicking intracellular environments. At physiological pH and pH 5.8, LP-IONPs did not release detectable levels of iron. However, at pH 4.5, up to 10% of the initial iron content was released within 1 h of incubation. This suggests that LP-IONPs, once internalized into cells, could partially release iron as the endolysosomal compartments acidify, but only under relatively highly acidic conditions. The release and increase in the distance between individual IONPs, along with their potential reduction in size due to partial dissolution, could explain the increase in *R*_1_ and decrease in *R*_2_.

Another possible explanation for the observed MRI shift is the entrapment of released IONPs in exocytotic vesicles. Kang et al. demonstrated that nanoparticles as small as 5 nm, like our IONPs, are substantially excreted by eukaryotic cells within 24 h.^55^ Although encapsulating IONPs within LP-IONPs might influence this process, further studies are required to confirm this hypothesis. To gain further insight, we performed high-angle annular dark-field (HAADF) TEM observations coupled with energy-dispersive X-ray spectroscopy (EDX) analyses of HepG2 cells after LP-IONP uptake at various time points. The results, shown in Figures 5E and S7, indicate that detectable iron accumulations were present at 6 and 24 h, potentially corresponding to liposomes still encapsulating IONPs within the cells. However, by day 3, these structures were no longer observed, likely due to partial IONP dissolution and excretion via exocytosis.

It is worth noting that the EDX limit of detection is typically 0.1% for bulk materials.^56^ Given the presence of other metals required to visualize cellular structures in these resin-embedded samples, IONPs are likely to be only detectable if they remain coconcentrated and relatively intact.

To investigate the photoinduced release of DOXO in cells, we initially performed cell viability studies with LP-IONP_DOXO_ loaded with a DOXO molar ratio of 0.03 ± 0.01 [DOXO]/ [phospholipid], at different phospholipid concentrations (0, 2, 20, 100, and 150 μM) and at different time points (4, 6, 24, and 48 h) (Figure 6). These studies revealed a significant decrease in cell viability at 24 h for phospholipid concentrations around 100 μM, with an even greater increase in cell death observed after 48 h.

**Figure 6:**
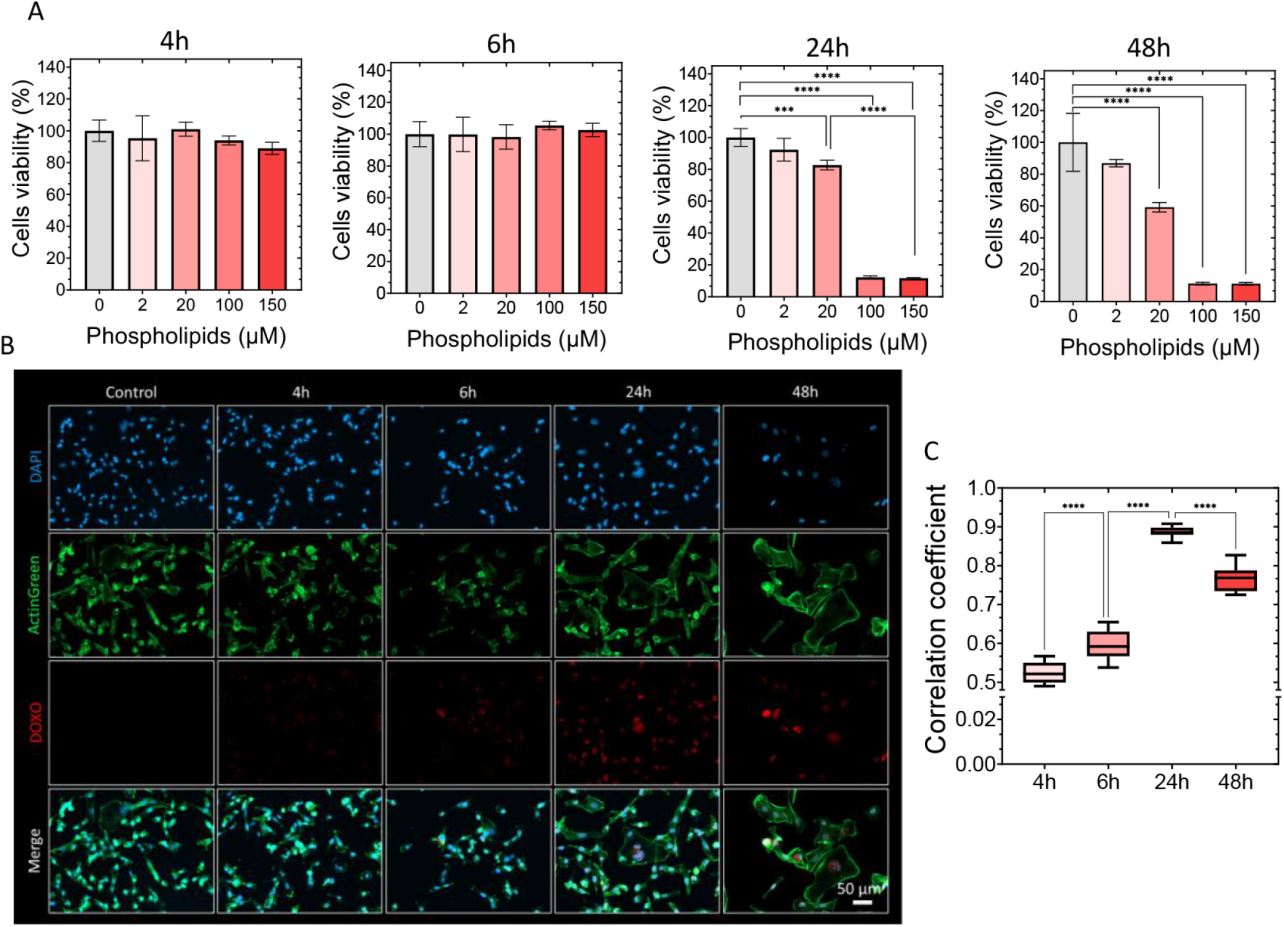
(A) Dose dependent cell viability in the MDA-MB-231 cell line incubated with LP-IONP_DOXO_ at four different time points (4, 6, 24, and 48 h). (B) Fluorescence images of the nucleus (DAPI, blue), cytoskeleton (ActinGreen, green), and DOXO (red). (C) Pearsońs correlation coefficient between DAPI and DOXO staining.

To confirm that DOXO reached the cell nucleus, where it exerted its effect by intercalating DNA base pairs and causing DNA damage, we performed confocal microscopy colocalization studies using DAPI staining of the cell nucleus and DOXO. The results, shown in Figure 6C, indicate that the highest level of colocalization occurred at 24 h, followed by a decrease, likely due to cell division, apoptosis, and subsequent loss of cellular structural integrity. Moreover, they confirmed cytosolic drug delivery of small molecules after 24 h, similar to that observed with DiOC_18_-loaded LP-IONP (Figure S5). We also analyzed free DOXO uptake and its colocalization with the cell nucleus using DAPI (Figure S8) and found that the highest correlation coefficient was achieved after 6 h of incubation. This suggests a delay in drug delivery from LP-IONP_DOXO_, likely caused by its encapsulation, LP-IONP_DOXO_ endocytosis, and subsequent cytosolic release, as reported elsewhere.

The observed time points for drug delivery and the dose-dependent effect shown in Figure 6 guided the selection of experimental conditions for studying the photothermal effects on both DOXO release and cell death induced by the irradiation of LP-IONP with NIR-II light.

Thus, we evaluated cell viability in MDA-MB-231 cells subjected to various treatments at different time points using calcein acetoxymethyl ester (calcein-AM) staining (Figure 7A). In viable cells, hydrolysis of calcein-AM by intracellular esterases generates green fluorescent calcein, which is retained in the cytoplasm. This fluorescence is rapidly lost under conditions that cause cell death, allowing us to monitor the effects of the different treatments applied: NIR-II irradiation only, incubation with empty LP-IONP, incubation with empty LP-IONP irradiated with NIR-II light, incubation with LP-IONP_DOXO_, and finally, incubation with LP-IONP_DOXO_ irradiated with NIR-II light. Figure 7A,B shows the results of this study, where cell viability was analyzed with calcein continuously from 2 to 7 h after the different treatments. As shown in Figure 7B, the highest reduction in viability, below 70%, was observed for cells administered with LP-IONP_DOXO_ and irradiated with NIR-II after 7 h of treatment. However, the effect of remote release of DOXO and the decrease in cell viability were visible 3 h after administration, in contrast to the other treatments, where a slight increase in cell proliferation was observed. Notably, under our working conditions, LP-IONP irradiation and consequent heat generation did not produce any effect on cell viability in that time interval (2–7 h posttreatment), so that the decrease in viability can be attributed to the controlled release of DOXO.

**Figure 7:**
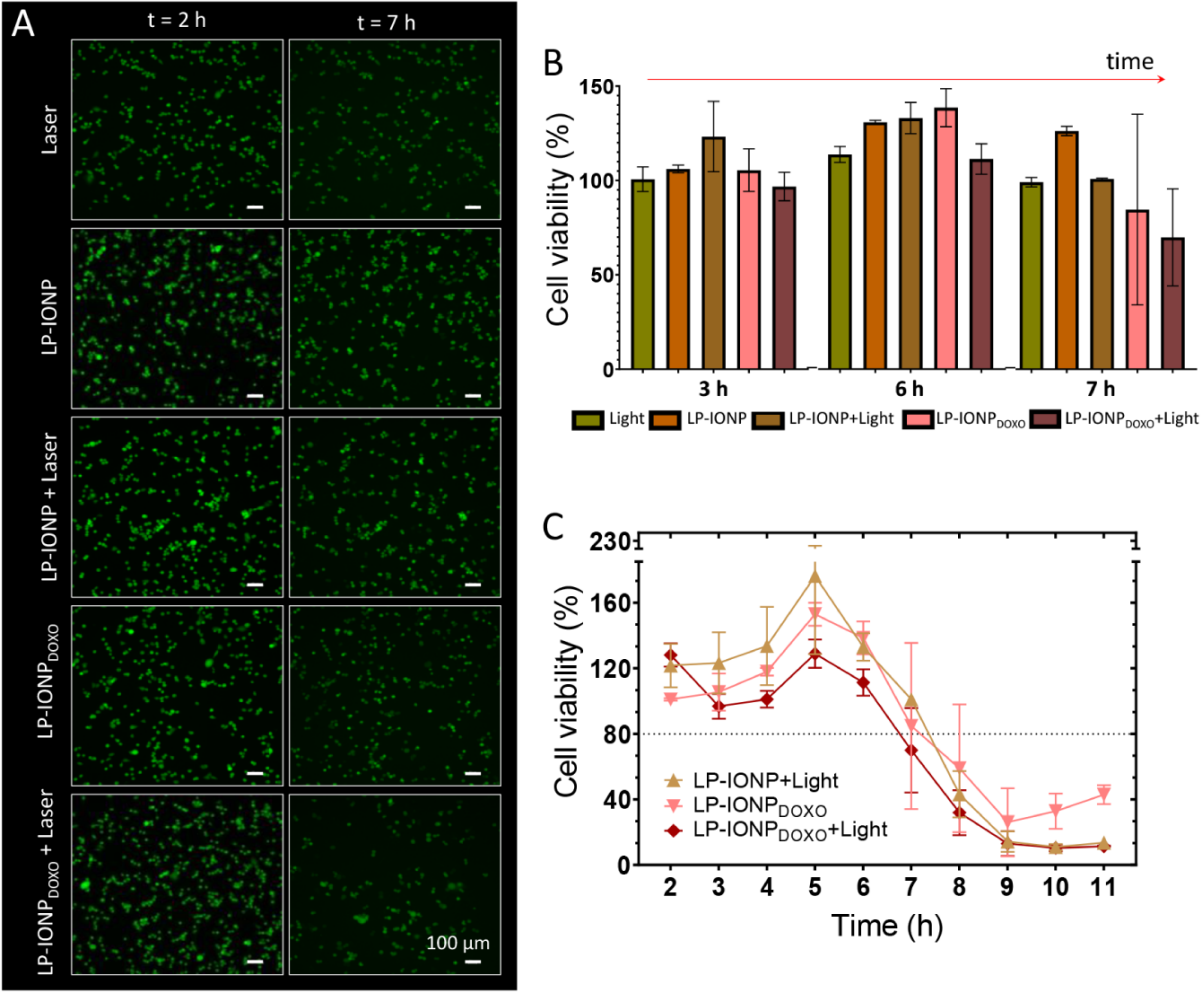
(A) Fluorescence microscopy images at 2 and 7 h of MDA-MB-231 cells stained with calcein AM, showing green fluorescence indicative of viable cells. Cells were incubated for 5 h with the corresponding magnetoliposomes at 0.2 μmol Fe/mL and images were taken after laser irradiation (1 W for 10 min). (B) Quantitative analysis of cell viability at 3-, 6-, and 7-h postlaser irradiation. (C) Time-dependent viability of MDA-MB-231 cells after NIR-II irradiation.

Since cell death by mild hyperthermia depends on several factors, such as the temperature reached intracellularly (40–45 °C), the specific cell line, and the time after exposure,^57^ we continued to measure the effect of the treatments for up to 11 h. In this case, the effect of heat generation of the cells treated with LP-IONP and NIR-II light could be observed, as cell viability was reduced, reaching almost 10% after this time. This reduction in cell viability was also observed for the combined treatment using NIR-II and LP-IONP_DOXO_ but was not pronounced when the treatment did not include NIR-II and was based on only LP-IONP_DOXO_. This suggests that LP-IONP could be used in both remote-controlled drug release and hyperthermia applications, and both could be more effective than treatments based solely on DOXO release.

Finally, *in vivo* studies were conducted to evaluate the biodistribution, safety profile, and integrity of LP-IONPs in mice using MRI and histological analysis (Figure 8). Following intravenous injection of LP-IONPs (1.5 mg Fe/kg), *T*_1_ and *T*_2_ maps were acquired at 3 and 24 h to evaluate both organ accumulation and the potential *T*_2_-*T*_1_ shift. The results obtained with *T*_2_ mapping analysis showed accumulation of LP-IONPs in the liver after 3 h (Figure 8B), consistent with the biodistribution of IONP-based contrast agents of similar size (50–200 nm).^58,59^ *R*_1_ measurements also showed a slight increase between baseline and 3 h, supporting the observations from the *R*_2_ data. Kidney analysis at 3 h postinjection showed no evidence of magnetic nanoparticle accumulation, suggesting that any free IONPs present in circulation were likely cleared by renal excretion by that time. Interestingly, at 24 h postinjection, a pronounced shift in the liver’s contrast behavior was observed, characterized by a marked decrease in *R*_2_ values and a modest increase in *R*_1_ values. Histological analysis confirmed the continued presence of LP-IONPs in the liver at this time point (Figure 8C), suggesting that the observed shift reflects a time-dependent *T*_2_-to-*T*_1_ transition in the relaxation properties of the particles, likely due to LP-IONP degradation similar to what we observed in cell cultures at longer times (Figure 5). The comparison of the temporal behavior of other magnetic nanoparticles also suggests this *T*_2_-to-*T*_1_ shift. For example, our group has previously demonstrated that nonencapsulated ultrasmall magnetic nanoparticles,^60^ accumulated in the liver and led to sustained *T*_2_ contrast beyond 24 h, while *T*_1_ contrast tended to recover after 24 h when the particles remained in the tissue, contrary to what we observed with LP-IONPs. In any case, this change in MRI contrast will have to be confirmed in other tissues. Histological analysis using hematoxylin and eosin (H&E) staining also revealed no significant structural abnormalities or organ damage in the kidney and liver tissues (Figure 8D).

**Figure 8:**
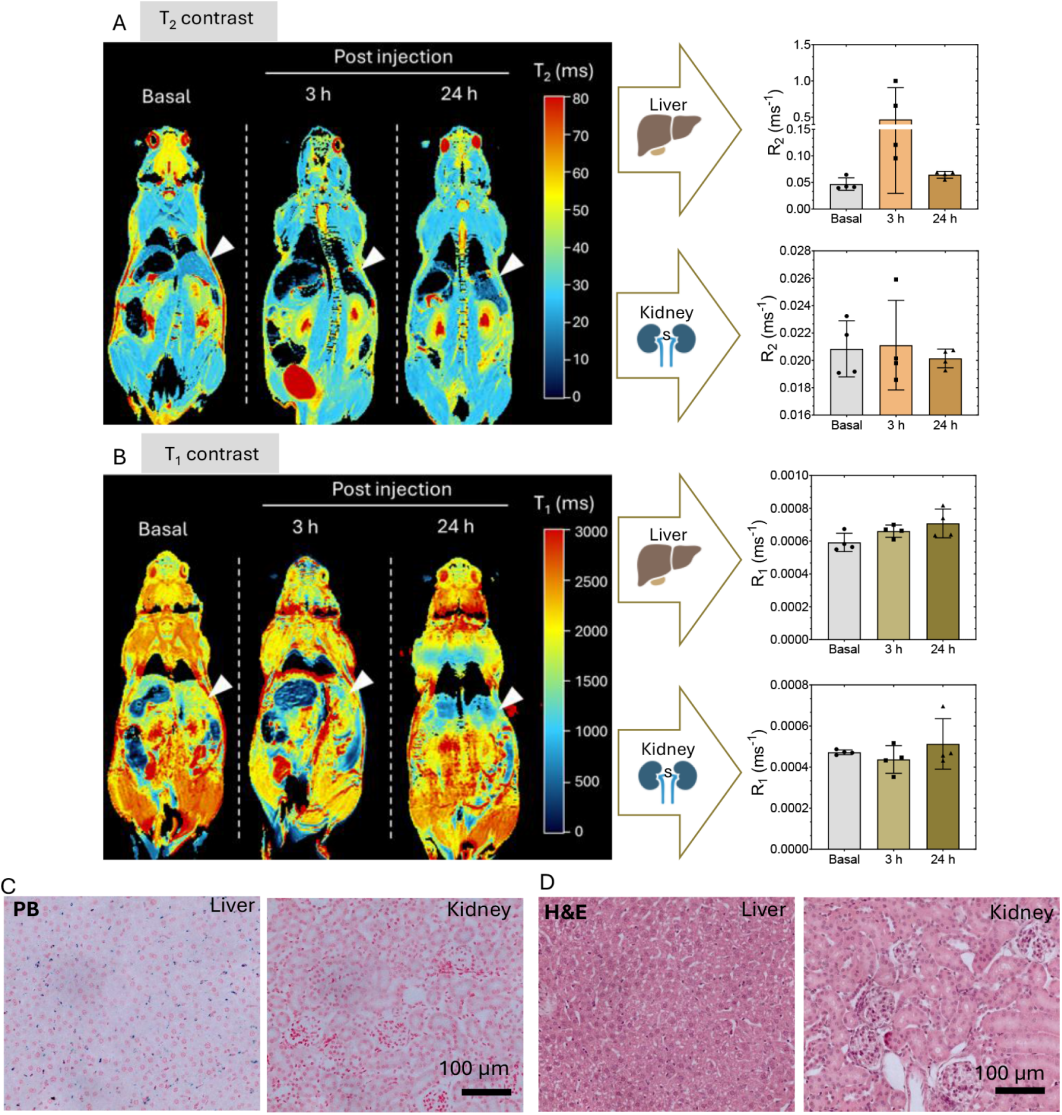
Representative *T*_2_ (A) and *T*_1_ (B) relaxation maps of the mouse body, along with the corresponding *R*_2_ (1/*T*_2_) and *R*_1_ (1/*T*_2_) values (*n* = 4), obtained from contrast analysis of the liver and kidneys before (baseline) and after intravenous administration of LP-IONP (at 3 and 24 h). The liver is indicated by white arrows. (C) PB staining of the liver and kidney 24 h after the administration of LP-IONP and the corresponding tissue stained with H&E (D). Scale bar 100 μm.

Future applications of LP-IONPs could explore MRI-guided interventions for targeted drug delivery using NIR-II-triggered remote release. Such approaches would be particularly advantageous for organs directly administered with LP-IONPs, where NIR-II irradiation can be applied either through a direct laser beam or via an optical fiber. Notably, drugs that exhibit release dynamics from magnetoliposomes slower than those of DOXO, which demonstrated rapid intracellular dispersion, may offer greater potential for controlled release using NIR-II irradiation.

Although our intracellular MRI findings suggest that LP-IONP degradation can be monitored, the release of DOXO or DiOC18 was not directly visualized, likely due to differences in size and chemical composition. Future studies are required to investigate the biotransformation of LP-IONPs in other organs and the release kinetics of encapsulated drugs following tissue accumulation. Collectively, our results underscore the promise of LP-IONPs for remotely controlled drug release, coupled with their efficacy as *in vivo* MRI contrast agents.

## 4 Conclusions

This study presents an innovative NIR-II photoresponsive and MRI contrast-responsive liposomal formulation that encapsulates colloidally stable, free-floating, and ultrasmall magnetic nanoparticles within the inner aqueous phase. This novel approach provides a versatile strategy for modifying the phospholipid composition through hydrophobic interactions, as demonstrated by the post incorporation of PEGylated phospholipids, which is important for future *in vivo* applications.

Due to the colloidal stability of the iron oxide nanoparticles encapsulated within the liposome, these hybrid nanomaterials exhibited unprecedented switchable MRI properties, transitioning between *T*_2_ and *T*_1_ contrasts upon liposome degradation or release of IONPs. This discovery paves the way for advanced imaging-guided drug release applications. Furthermore, this study demonstrates that confining ultrasmall IONPs within the liposome enhances their photothermal activity, enabling the precisely controlled release of a model drug upon NIR-II irradiation.

Comprehensive *in vitro* and *in vivo* studies, as well as MRI and histology, confirmed the formulation’s safety profile, biodistribution, and multifunctional properties.

## Supplementary Material



## Notes

Physicochemical properties of empty LPs and LP-IONPs, colloidal stability assay, influence of temperature increase on the hydrodynamic ratio and the corresponding percentage of drug release, UV–vis-NIR spectra, cell viability assays, MRI contrast measurements of control samples, TEM and HAADF images of cell cultures, dye colocalization analysis, effect of DOXO release on cell viability in the absence of NIR-II, and experimental information about in vivo experiments (PDF)
