# Supplementary material for: NIR-II Photoresponsive Magnetoliposomes for Remote-Controlled Release and Magnetic Resonance Imaging
Source: ACS Appl Bio Mater. 2025 May 22;8(6):4855–69. doi: 10.1021/acsabm.5c00233 (PMC12509320; doi:10.1021/acsabm.5c00233)
Supplement: Supplementary file 1 [file mt5c00233_si_001.pdf]

Supporting Information

NIR-II photoresponsive magnetoliposomes for remote controlled release and magnetic resonance imaging

Laura Fernández-Méndez,[1,2] Yilian Fernández-Afonso[3,4], Pablo Martínez-Vicente[4,5], Ainhize Urkola-Arsuaga[1], Claudia Miranda-Pérez de Alejo, [1,2] Irati L. de la Pisa[1,2], Sandra Plaza-García[1], Jesús Ruíz-Cabello[1,6,7,8], Pedro Ramos-Cabrer[1,6]*, Lucía Gutiérrez[4,9]*, Susana Carregal-Romero[1,6,7]*

[1]Center for Cooperative Research in Biomaterials (CIC biomaGUNE), Basque Research and Technology Alliance (BRTA), 20014, Donostia, Spain

[2]Euskal HerrikoUnibertsitatea (UPV/EHU), 20018 Donostia, Spain

[3]Instituto de Ciencia de Materiales de Madrid (ICMM/CSIC), 28049 Madrid, Spain.

[4]Instituto de Nanociencia y Materiales de Aragón (INMA), CSIC-Universidad de Zaragoza, 50018 Zaragoza, Spain

[5]Departamento de Bioquímica y Biología Molecular y Celular. Universidad de Zaragoza, 22002 Huesca, Spain

[6]Ikerbasque, Basque Foundation for Science Ikerbasque, 48013 Bilbao, Spain

[7]Centro de investigación en red de enfermedades respiratorias (CIBERES). Instituto de Salud Carlos III, 28029 Madrid, Spain

[8]Departamento de Química en Ciencias Farmacéuticas. Universidad Complutense de Madrid, 28040 Madrid, Spain

[9]Centro de Investigación Biomédica en Red de Bioingeniería, Biomateriales y Nanomedicina (CIBER-BBN), 50019 Zaragoza, Spain.

*Corresponding author's email: pramos@cicbiomagune.es, lu@unizar.es, scarregal@cicbiomagune.es

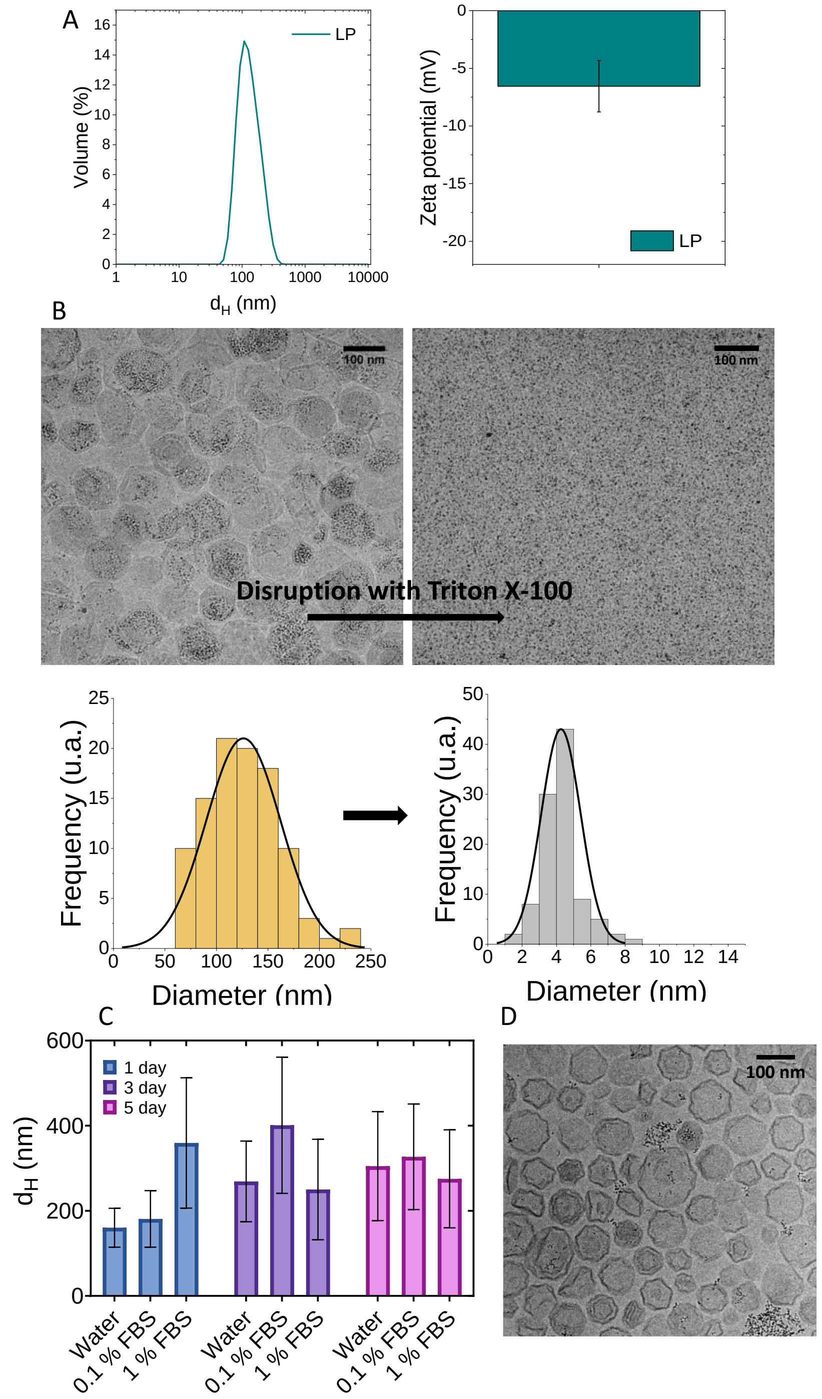

Figure S1. A) Hydrodynamic diameter (d_H) and zeta-potential of empty LP (based on DPPC:Lyso-PC). B) CryoTEM images of LP-IONP before and after disruption with Triton X-100 and the corresponding diameter of LP-IONP and IONP measured from TEM images. C) Colloidal stability of LP-IONP in water and 0.1 and 1% FBS solution. D) CryoTEM image of the failed encapsulation of IONP when DSPE-PEG is added during the formation of phospholipid thin-film prior to extrusion.

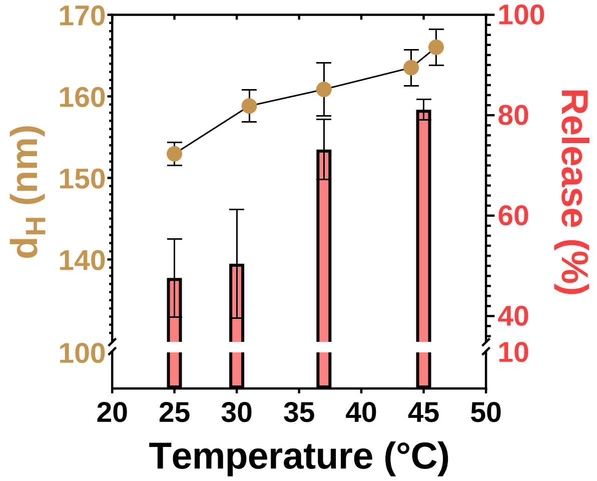

Figure S2. Influence of temperature on the hydrodynamic diameter (d_H) and drug release in water of LP-IONP$_{DOXO}$ after 10 minutes of incubation.

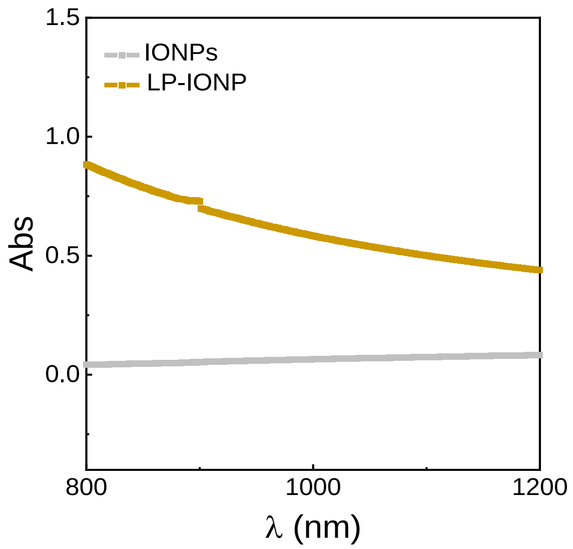

Figure S3. UV-Vis-NIR spectra at 0.6 mg Fe/ mL in the range of 800–1200 nm of IONP and LP-IONP

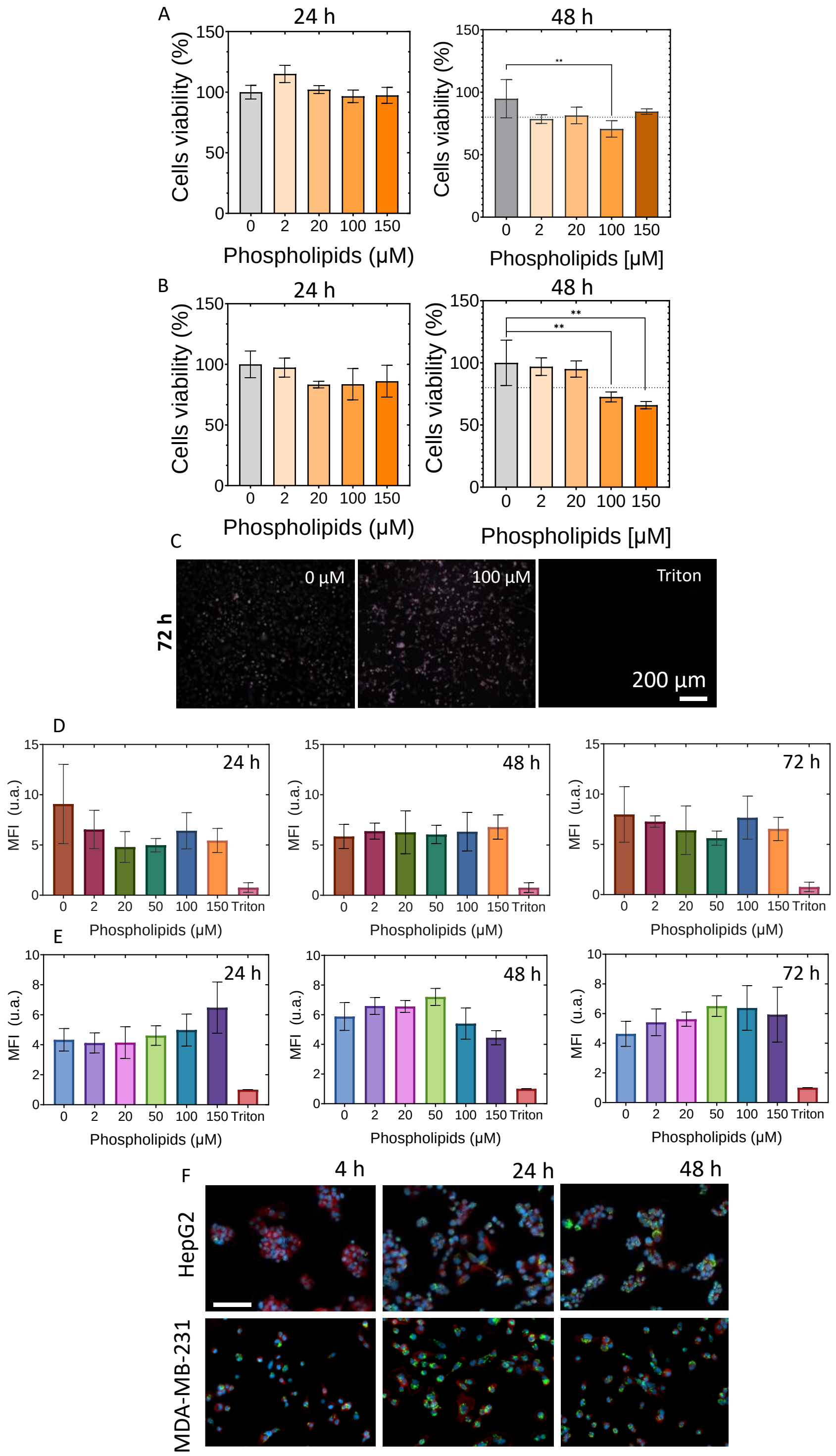

Figure S4. Cell viability after 24 and 48 hours of incubation with LP-IONP in HepG2 cells (A) and in MDA-MB-231 cells (B) measured by MTT assay. (C) Representative confocal image of MDA-MB-231 cells incubated with 100 µM LP-IONP (based on phospholipid concentration) for 72 hours, stained with ThiolTracker^{TM} Violet (λ_{em}= 525 nm). (D) Fluorescence measurements of the intracellular thiol probe ThiolTracker^{TM} Violet in MDA cell cultures incubated at 24, 48 and 72 hours at different concentrations of LP-IONP in HepG2 (D) and MDA-MB-231 cells (E). (F) Cellular uptake of LP-IONP (at 20 µM, labeled with $DiOC_{18}$) over time in HepG2 and MDA-MB-231 cells.

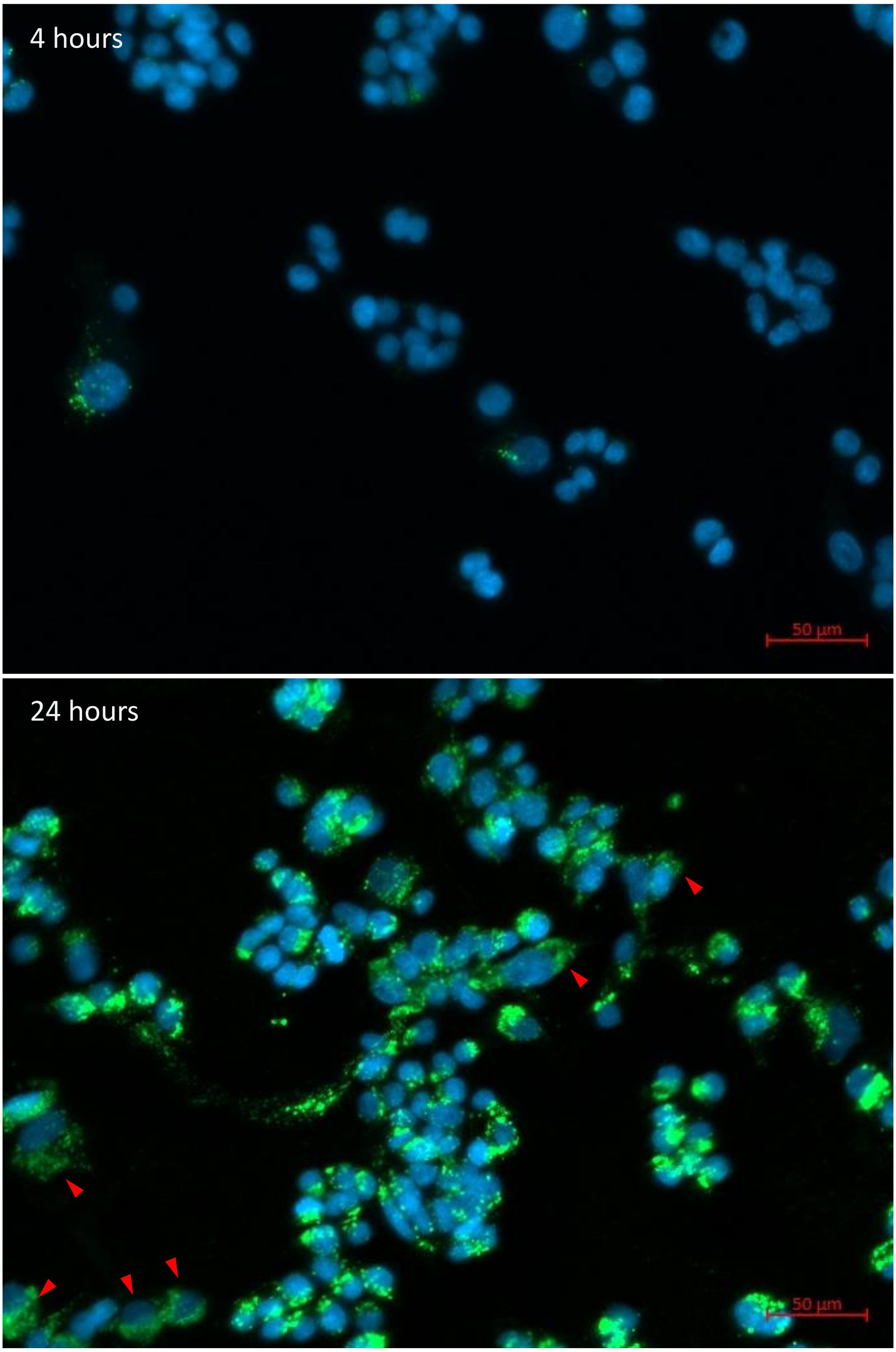

Figure S5. Confocal images of LP-IONPs loaded with $DiOC_{18}$ after 4 and 24 hours of incubation with HepG2 cells. After 24 hours, several cells (highlighted with red triangles) showed cytosols homogeneously stained with the green dye, potentially indicating its release from the LP-IONPs. The nucleus was stained with the blue dye DAPI.

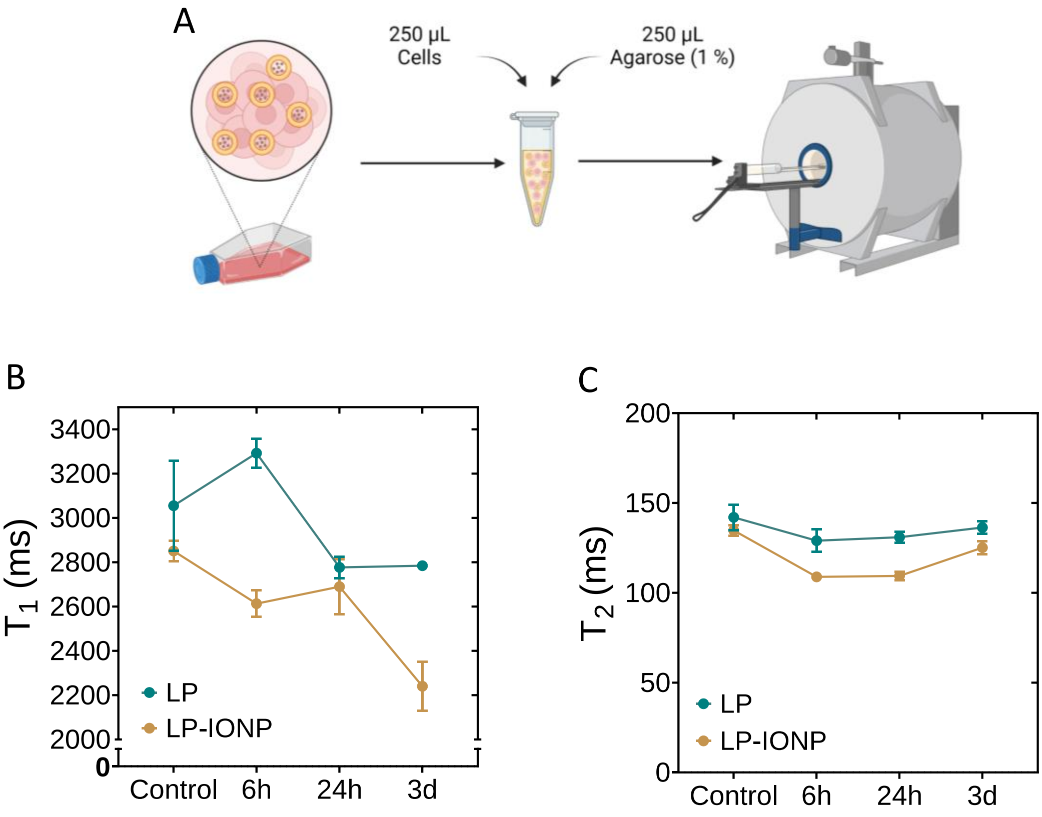

Figure S6. (A) Schematic representation of the cell preparation procedure for MRI measurements. (B) T_1 and (C) T_2 values of empty LP (green) and LP-IONP (brown) at 6 hours, 24 hours, and 3 days.

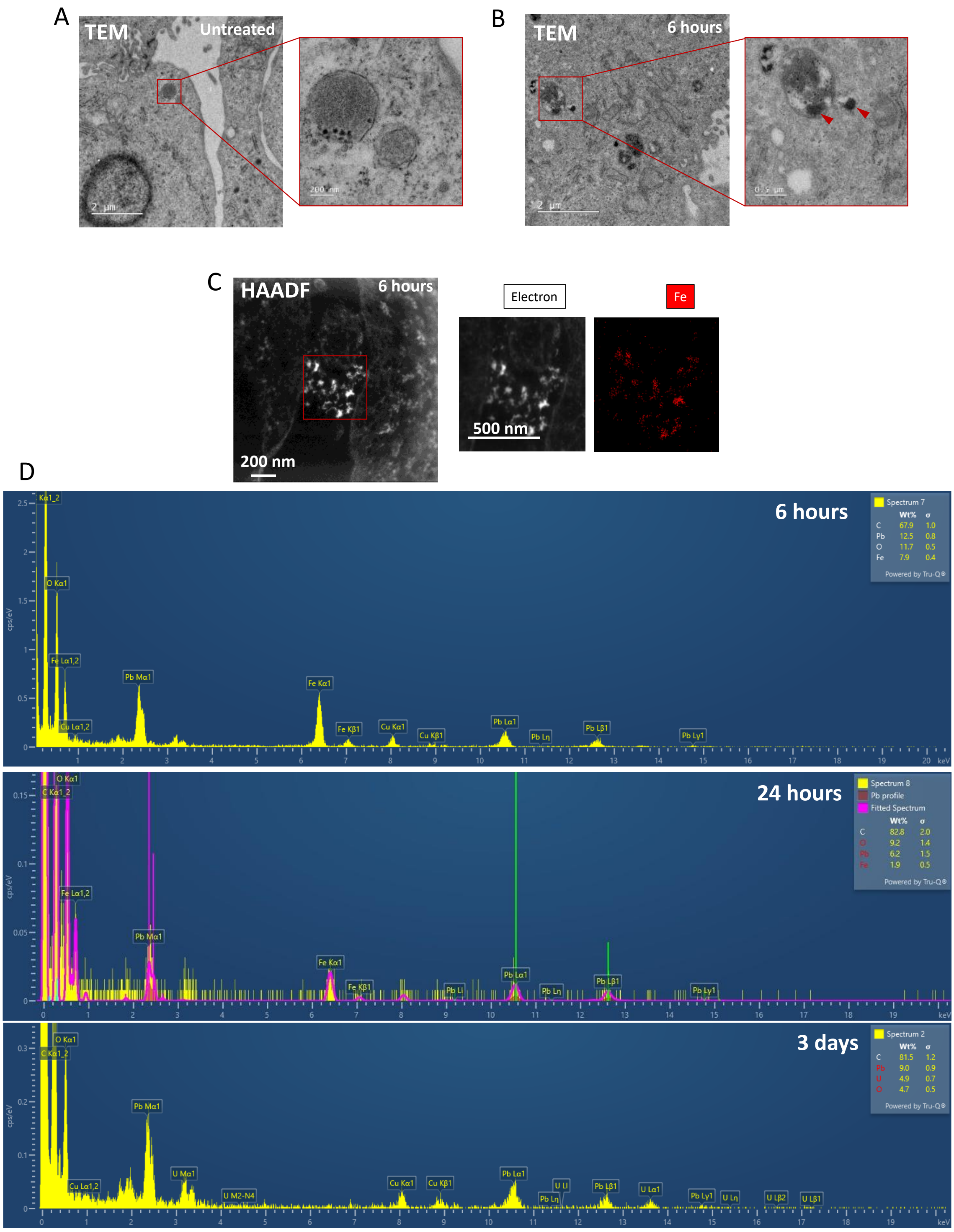

Figure S7. (A) Representative TEM images of HepG2 cells and (B) representative TEM and (C) HAADF images of HepG2 cells incubated for 6 hours with LP-IONP, along with the corresponding EDX image showing the Fe element in the regions of interest highlighted by red squares. (D) EDX spectra of HepG2 cells incubated with LP-IONPs at different time points, along with the corresponding elemental percentages of Fe: 7.9% at 6 hours, 1.9% at 24 hours, and 0% at 3 days.

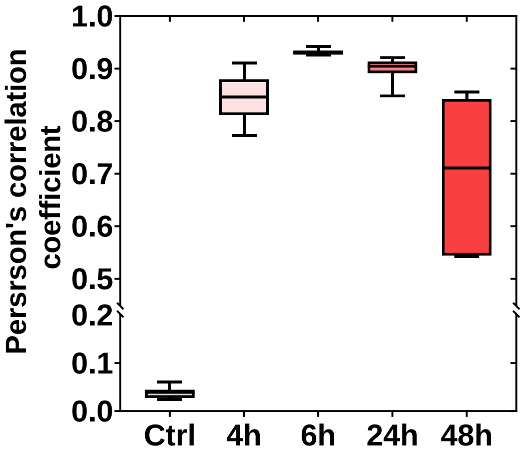

Figure S8. Time-dependent co-localization of free DOXO and DAPI in MDA-MB-231 cells.

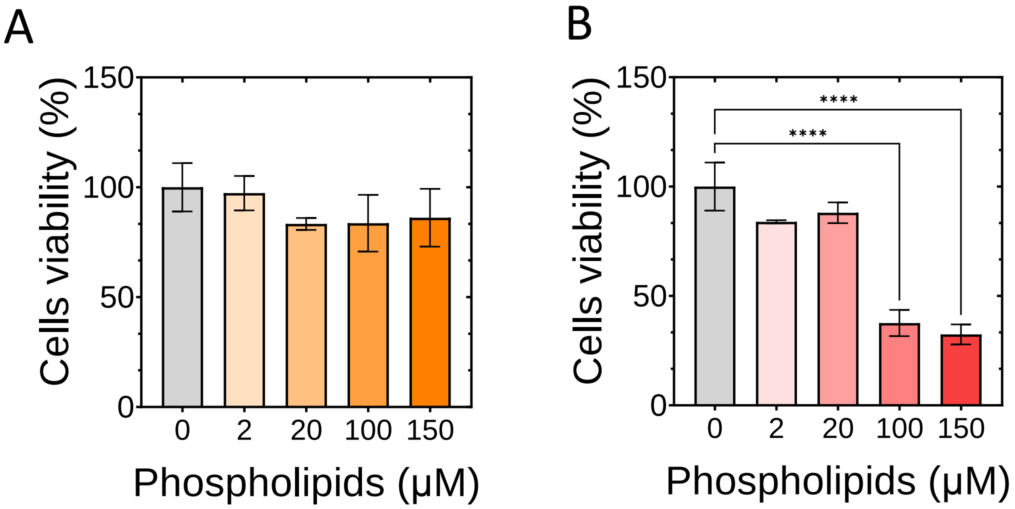

Figure S9. Influence of phospholipid concentration on the cell viability of HepG2 cells incubated with LP-IONP (A) and LP-IONP$_{DOXO}$ (B) after 24 hours.

Biodistribution of LP-IONP *in vivo.* 11-week-old female C57BL/6JRj mice (n=4) were imaged prior to administration to obtain reference scans. LP-IONPs (1.5 mg/kg) were then injected intravenously into the mice via the tail vein in 100 µL doses. Abdominal MR images were then acquired using a T1- and T2-weighted sequence at different time points (basal, 3 and 24 hours). All experiments were performed on a 7 Tesla Bruker Biospec 70/30 USR MRI system (Bruker Biospin GmbH, Ettlingen, Germany) connected to an AVANCE III console. A BGA12 imaging gradient system (maximum gradient

strength 400 mT/m) with a 39 mm diameter Neos Whole Body Mouse transmit/receive coil was used for MRI data acquisition.

Anatomical images of the abdomen were acquired using a Bruker gradient-echo FLASH sequence with the following parameters: TE 4 ms, respiration synchronized (TR 600 ms), FA 30, 2 averages, 182 x 182 points, a field of view of 3.0 cm x 3.0 cm, and 10 non-contiguous slices with a slice thickness of 1.0 mm. T_1 maps were obtained by spin echo saturation recovery using a variable repetition time Bruker RAREVTR technique. Images were acquired at 8 different TR values (6500, 3000, 1500, 1000, 800, 600, 450, 310 ms), effective TE 7 ms, RARE factor 2, 2 averages, 320 x 160 points, a field of view of 8.0cm x 4.0cm, 14 slices with a slice thickness of 1.0mm and a slice gap of 0.25 mm. T_2 maps were obtained using a multi-slice spin echo (MSME) sequence. The TE values were varied in 30 steps from 9 ms to 270 ms and the breathing was synchronized (TR 1600 ms). The acquisition was performed with fat suppression, 1 average, 320 x 160 points, a field of view of 8.0 cm x 4.0 cm, 14 slices with a slice thickness of 1.0 mm and a slice gap of 0.25 mm. Quantification for liver and kidney was performed using T_1 and T_2 maps. The final T_1 and T_2 maps were finally analyzed using ImageJ 1.53t software.

Histology. Organ sections (4 µm thick) embedded in paraffin and affixed to glass slides were stained with hematoxylin and eosin (H&E) and Prussian blue (PB) using standard histological protocols to assess histopathological lesions and iron localization. Images were captured with a Cell Axio Observer Microscope and analyzed using ImageJ software.
